# Tailored Spin Coupling of Single-Molecule Magnets with a Single Charge-Density-Wave Metal Layer

**DOI:** 10.1021/jacs.5c21952

**Published:** 2026-03-12

**Authors:** Can Zhang, Fudi Zhou, Heng Jin, Lili Zhou, Zhaoteng Dong, Mengya Ren, Quanzhen Zhang, Huixia Yang, Xiaolong Xu, Yuan Xiao Ma, Lan Chen, Thomas A. Jung, Bing Huang, Hong-Jun Gao, Yu Zhang, Yeliang Wang

**Affiliations:** † School of Integrated Circuits and Electronics, MIIT Key Laboratory for Low-Dimensional Quantum Structure and Devices, 47833 Beijing Institute of Technology, Beijing 100081, China; ‡ School of Interdisciplinary Science, State Key Laboratory of Environment Characteristics and Effects for Near-space, Beijing Institute of Technology, Beijing 100081, China; § School of Physics, University of Electronic Science and Technology of China, Chengdu 610054, China; ∥ 298288 Beijing Computational Science Research Center, Beijing 100193, China; ⊥ 47849 Institute of Physics, Chinese Academy of Sciences, Beijing 100190, China; # School of Physical Sciences, University of Chinese Academy of Sciences, Beijing 100190, China; ∇ Songshan Lake Materials Laboratory, Dongguan 523808, China; ¶ Laboratory for X-ray Nanoscience and Technologies, Paul Scherrer Institut (PSI), 5232 Villigen, Switzerland; □ Key Laboratory of Multiscale Spin Physics (Ministry of Education), Beijing Normal University, Beijing 100875, China

## Abstract

The interplay between spin and charge can give rise to remarkable quantum states of matter. A celebrated example is the Kondo effect, which occurs when localized magnetic impurities are screened by itinerant electrons. While significant advances have been made in probing the Kondo effect in systems consisting of magnetic impurities adsorbed on conventional bulk metals, its manifestation on unconventional metals with strong many-body interactions, particularly down to atomic-layer thickness, has hitherto remained unexplored. Here we investigate the charge and spin interaction between magnetic cobalt phthalocyanine (CoPc) molecules acting as spin-bearing Kondo impurities and a single, substrate supported layer of the charge-density-wave (CDW) metal H-NbSe_2_. Remarkably, we present unambiguous Kondo signatures on certain adsorption sites. We identify four distinct configurations depending on the position of the Co^2+^ ion relative to the atomic lattice and the CDW superlattice of NbSe_2_. We show precise control of the Kondo effect by reversible repositioning of the CoPc molecule between the different positions. Our scanning tunneling microscopy (STM) and spectroscopy (STS) measurements further reveal the symmetry breaking of the Kondo resonance, indicating a pronounced magnetic anisotropy in CoPc/NbSe_2_. More importantly, this interaction can induce local magnetism into the nonmagnetic NbSe_2_ layer, offering new possibilities for tailoring spin textures.

## Graphical abstract

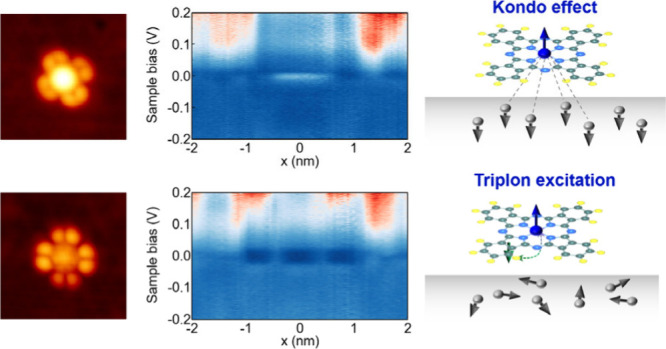

## Introduction

The interaction between spin and charge in condensed matter systems offers a fertile ground for the emergence of intriguing quantum phenomena,^1–5^ which can be exploited for a broad range of applications in spintronics, information storage, and quantum computing.^6–8^ A prototypical example is the Kondo effect, which arises when localized magnetic impurities are screened by surrounding itinerant electrons, thus causing a change in conductance.^9–11^ The ability to tune the Kondo effect would be valuable for controlling conductance through spin manipulation. Magnetic impurities absorbed on a metal surface are particularly outstanding in this regard, as their spin can be easily manipulated and precisely detected through scanning tunnelling microscopy (STM). Over the past few decades, substantial progress has been made in investigating the Kondo effect, primarily by the increased resistance caused by magnetic impurities in traditional three-dimensional (3D) bulk metals such as Au, Ag, and Cu and then by local tunneling spectroscopy at atomic and molecular adsorbates on elsewise atomically clean metal surfaces.^12–16^ More recently, attention has turned to magnetic impurities hosted on multilayer van der Waals (vdW) materials including metallic transition metal dichalcogenides (TMDs)^17–22^ as well as semimetallic Sb(111) and Bi(111).^23,24^ Notably, for the Kondo physics in these systems, the metallic materials mainly serve as reservoir of itinerant electrons.

The manifestation of the Kondo physics induced by unconventional metals with pronounced many-body interactions, particularly down to the atomic-layer thickness, has hitherto remained unexplored. A crucial step in this quest is to elucidate how molecular spins couple with electrons in many-body ground states, thereby mediating intricate new modes of interplay between charge and spin degrees of freedom. Generally, many-body electronic ground states are highly sensitive to the spatial dimensionality, where a reduced dimensionality often favors the emergence of exotic quantum states.^25^ The layered TMD material 2H-NbSe_2_ serves as a model metallic system in this regard. As the dimensionality reduced from bulk to monolayer, the in-plane inversion symmetry of NbSe_2_ is broken, giving rise to spin-momentum locking. Furthermore, monolayer NbSe_2_ exhibits a significantly enhanced charge density wave (CDW) order and suppressed superconductivity, driven by the interaction among ionic charge transfer, electron–phonon coupling, and electron correlation.^26–30^

Previous research has focused on CoPc molecules on superconducting bulk 2H-NbSe_2_, observing two distinct spin states.^20^ However, while bulk NbSe_2_ exhibits a quasi-2D electronic structure due to weak interlayer vdW coupling, the spin states of CoPc on monolayer NbSe_2_ cannot be simply extrapolated from the bulk case. The reduced dimensionality profoundly alters the electronic properties of NbSe_2_, leading to distinct coupling behaviors with CoPc. Unlike bulk NbSe_2_, which can be treated as a relatively uniform metal, monolayer NbSe_2_ behaves more like a spatially inhomogeneous metal with a periodic CDW potential. Additionally, the reduced Coulomb screening in monolayer NbSe_2_ enhances electron–electron correlations, facilitating stronger charge transfer and spin coupling with CoPc (Table S1). These unique features make monolayer NbSe_2_ an ideal platform for exploring Kondo physics and broader spin-charge interactions in many-body quantum systems.

In this work, we investigate the spin coupling in systems consisting of magnetic impurities on monolayer TMD metals with a CDW ground state by depositing magnetic cobalt phthalocyanine (CoPc) molecules onto monolayer H-NbSe_2_. Our high-resolution STM measurements reveal four distinct molecular configurations, determined by the relative position of the Co^2+^ ion with respect to the atomic lattice and the CDW superlattice of NbSe_2_. In our in-depth study below, we find that these configurations can be reversibly switched through molecular repositioning, thereby enabling targeted modulation of the CoPc spin states and allowing the Kondo effect to be toggled on and off. Furthermore, the Kondo resonance map exhibits a pronounced 2-fold symmetry in real space, indicating the magnetic anisotropy in CoPc/NbSe_2_. Simultaneously, the monolayer NbSe_2_ can be locally magnetized. These findings shed new light on the CDW-involved Kondo effect, paving the way for tuning spin states and engineering complex topological spin textures.

## Results and Discussion

In our experiment, high-quality monolayer H-NbSe_2_ films are prepared on bilayer graphene (BLG)/SiC(0001) substrates, where the atomic lattice of NbSe_2_ is well resolved, and the charge density distribution exhibits a periodic modulation consistent with the CDW superlattice, as shown in Figure S1. We then sublime a submonolayer coverage of spin-bearing CoPc molecules (Figure 1a,b, Methods, and Figure S2).^31–33^ The adsorption of CoPc molecules slightly perturbs the topographic uniformity of the monolayer H-NbSe_2_, causing weak height fluctuations in the STM image. Nevertheless, this perturbation has a negligible effect on the local potential of the monolayer H-NbSe_2_, maintaining a well-defined periodic modulation of the charge density distribution, again consistent with the CDW superlattice of H-NbSe_2_, as shown in Figure S3.

**Figure 1:**
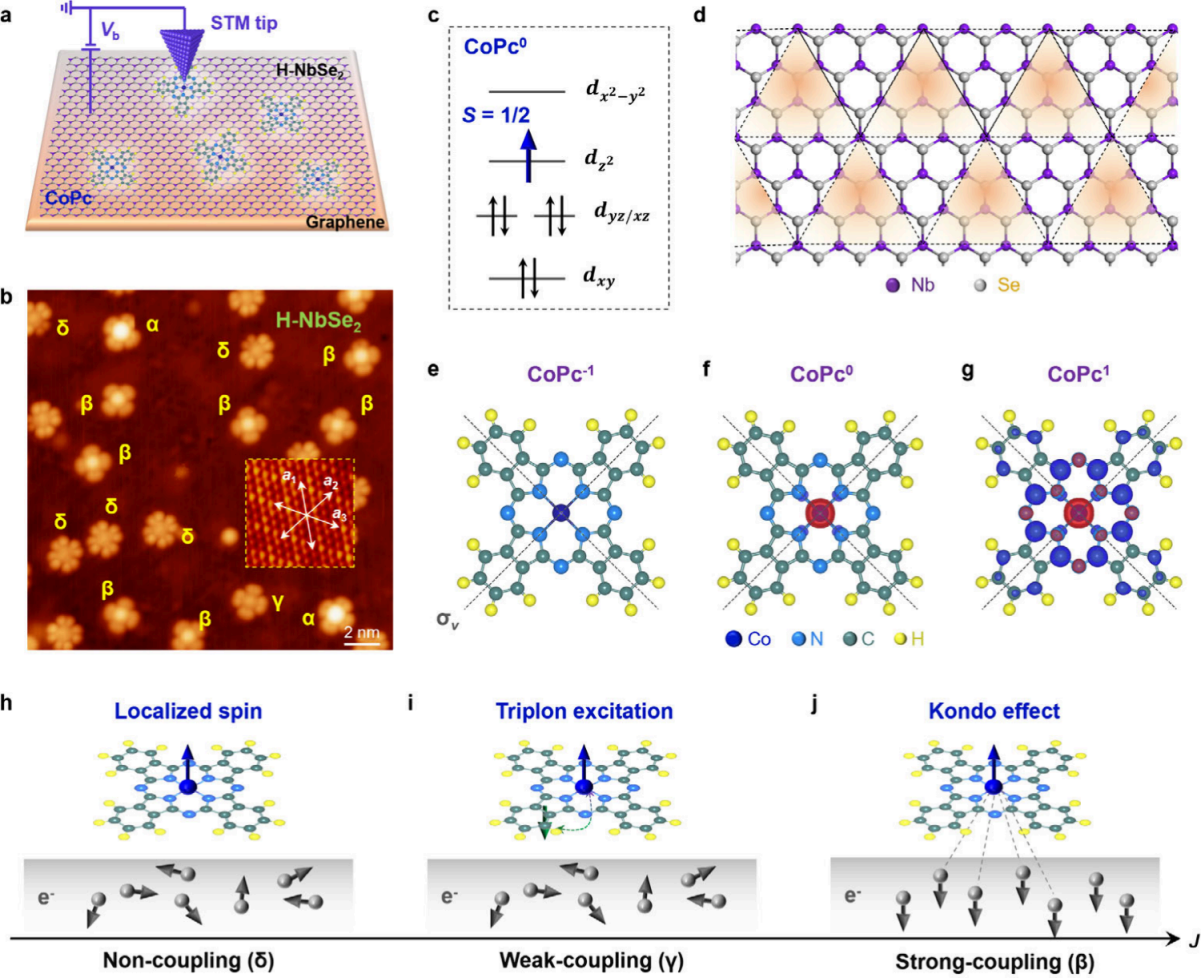
Structural and electronic properties of CoPc molecules on monolayer H-NbSe_2_. (a) Schematic sample structure and the experimental setup. Monolayer H-NbSe_2_ is first synthesized on BLG/SiC(0001) substrates, followed by the deposition of CoPc. (b) Large-scale STM image of CoPc on monolayer H-NbSe_2_ (*V*_*b*_ = −1.0 V, *I*_*t*_ = 10 pA). The four distinct configurations are labeled as α, β, γ, and δ. Inset: High-resolution STM image of monolayer H-NbSe_2_, with the lattice orientations marked by double-headed arrows. (c) Energy level of CoPc in the gas phase: One unpaired electron in the d*z* ^2^ orbital gives rise to a *S* = 1/2 spin. (d) Atomic structure of monolayer H-NbSe_2_. The top Se atoms form alternating higher and lower triangular motifs, following the 3 × 3 CDW periodicity. The higher motifs are marked by yellow shadows. (e-g) Calculated spin distributions of CoPc in negative (CoPc^–1^), neutral (CoPc^0^), and positive (CoPc^1^) charge states. The red/blue clouds represent the spin up/down electrons. The charge density isosurface is 0.002e/Å^3^. The molecular axes σ_v_ are indicated by dashed lines. (h–j) Schematic of charge and spin interactions between a localized magnetic moment in CoPc and itinerant electrons in monolayer H-NbSe_2_ under different coupling strengths.

A CoPc molecule comprises a planar metal–organic complex consisting of a central Co^2+^ ion surrounded by four Pc ligands, exhibiting D_2h_ symmetry. In the gas phase, an individual neutral CoPc molecule (CoPc^0^) endows an *S* = 1/2 spin,^34^ which is originated from an unpaired electron residing in the d*z* ^2^ orbital of the Co^2+^ ion^35^ (Figure 1c,f). Figure 1d presents an atomic model of monolayer H-NbSe_2_, where a Nb layer is sandwiched between two Se layers, with each Nb atom located within a trigonal prismatic cage formed by six nearest-neighbor Se atoms, resulting in C_3v_ symmetry. As the temperature drops to approximately 145 K, monolayer NbSe_2_ undergoes a 3 × 3 CDW transition, accompanied by atomic distortion where the top Se atoms form alternating higher and lower triangular motifs.^26–29^ Therefore, the deposition of CoPc molecules onto monolayer NbSe_2_ provide an ideal platform for precisely tuning the spin states of CoPc (Figure 1e-g) and tailoring the spin coupling between a local magnetic moment and a metal under the CDW ground state (Figure 1h-j).

Large-scale STM image shown in Figure 1b reveals that CoPc molecules on monolayer NbSe_2_ surfaces prefer to be sparsely distributed, with each molecule exhibiting a roughly cross-like appearance. High-resolution STM images further reveal that the apparent topography of CoPc on monolayer NbSe_2_ displays four different configurations, which can be categorized into α, β, γ, and δ (Figure 2a-d). At a given negative voltage, for the α and β configurations, the CoPc molecules typically display a pronounced central protrusion with a four-lobe pattern (Figure 2a,b). In contrast, the γ and δ configurations show a dip at the center, accompanied by an eight-lobe pattern (Figure 2c,d). The variation in brightness of the central Co^2+^ ion presented in STM images (Figure 2f,g) indicates distinct energies of the *d*_*Z*_^2^ orbital with respect to the Fermi level for these four configurations.

**Figure 2:**
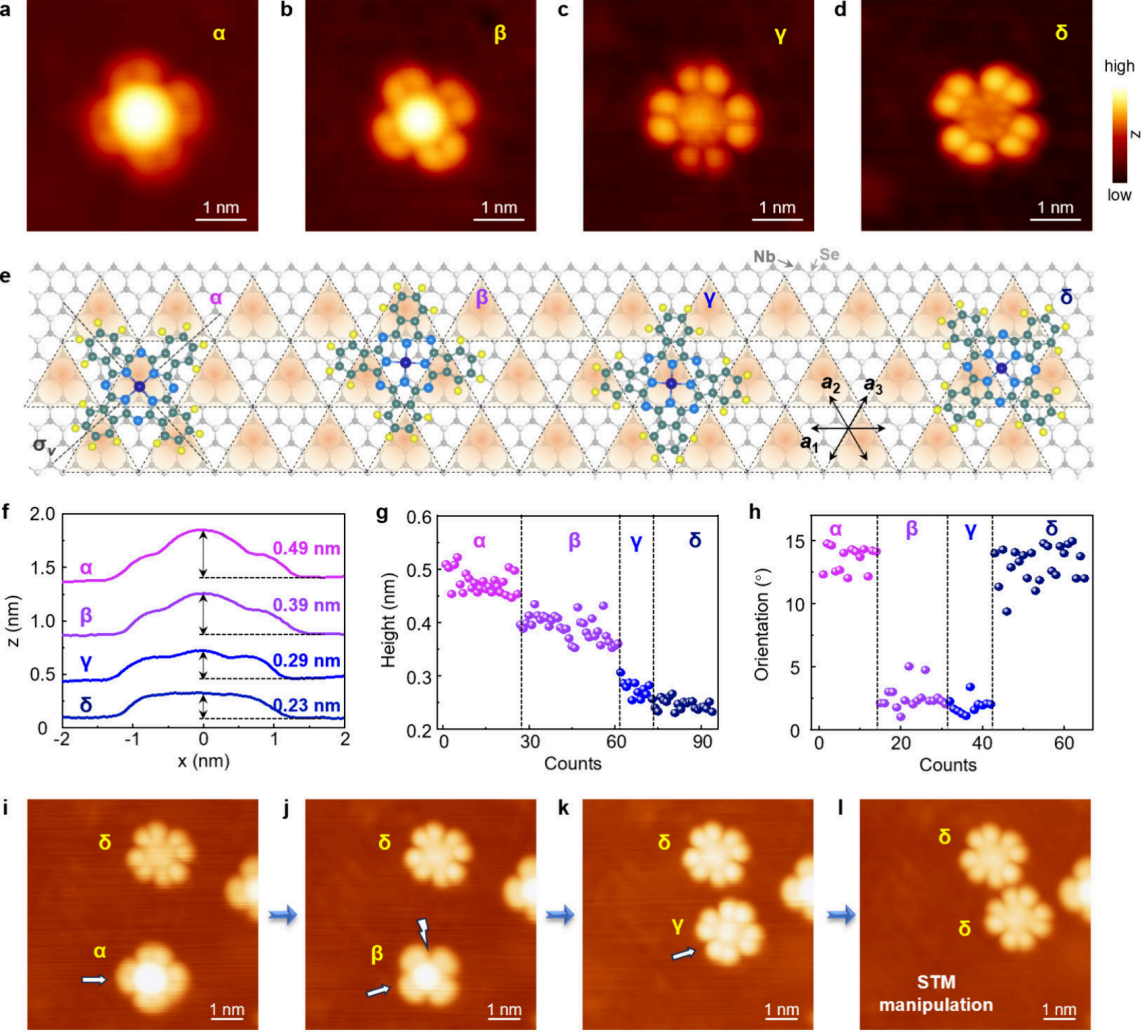
Characterization of CoPc molecules on monolayer H-NbSe_2_. (a–d) STM images of CoPc molecules on monolayer H-NbSe_2_ in the α, β, γ, and δ configurations, respectively (*V*_*b*_ = −1.0 V, *I*_*t*_ = 10 pA). (e) Atomic model of CoPc molecules relative to the underlying H-NbSe_2_ lattice, extracted from STM images. (f) STM height profiles measured across CoPc in the α, β, γ, and δ configurations at −1.0 V, revealing heights of approximately 0.49, 0.39, 0.29, and 0.23 nm, respectively. (g) Statistical distribution of apparent STM heights at the center of CoPc molecules on monolayer H-NbSe_2_. (h) Orientation of the σ_v_ axis in CoPc with respect to the NbSe_2_ lattice directions, showing a misalignment of ∼13° for the α and δ configurations and ∼2° for the β and γ. (i-l) STM micrographs taken during a repositioning sequence (*V*_*b*_ = −1.0 V, *I*_*t*_ = 10 pA). The arrow indicates the direction of the CoPc movement using the tip manipulation, while the lightning symbol represents the location where a transition has been induced via a tip pulse of 2 V.

Generally, to achieve maximum symmetry, one of the molecular axes σ_v_ in CoPc should be either parallel or perpendicular to the crystallographic axes of NbSe_2_ (double-headed arrows in Figure 1b). Due to the hexagonal, nonrectangular symmetry of NbSe_2_, the σ_v_ axes is expected to exhibit a misalignment of 0° or 15° relative to the NbSe_2_ lattice, which has been observed in systems where CoPc molecules are placed on bulk NbSe_2_ surfaces.^20^ However, contrary to this expectation, the σ_v_ axes in α-CoPc and δ-CoPc show an ∼13° misalignment relative to the atomic/CDW orientations of monolayer NbSe_2_, whereas an ∼2° misalignment is observed for the β and γ configurations (Figure 2h). This result highlights that the enhanced CDW order in monolayer NbSe_2_ modulates and stabilizes these four configurations.

A closer inspection also reveals that the central Co^2+^ ion in CoPc is positioned directly on the topmost Se atoms of monolayer NbSe_2_ for the α, β, and γ configurations, whereas it is offset from the Se atoms for the δ (Figure S4). When further considering the CDW superlattice of monolayer NbSe_2_, the Co^2+^ ion resides on the higher triangular motifs for the α and γ configurations, while on the lower motifs for the β and δ (Figure S5), as schematically illustrated in Figure 2e. Moreover, these four configurations can be controllably transformed through STM tip manipulation (Figure 2i-l). As moving the sites and orientation of CoPc with respect to the underlying NbSe_2_, transformation can occur between the α and β configurations, as well as between the γ and δ configurations. Transformation between the β and γ configurations, however, usually occurs by applying a tip pulse. By examining dozens of manipulating sequences, we find that all four configurations can be reversibly interconverted by adjusting the position and/or orientation of CoPc on NbSe_2_ (Figure S6), which help us exclude hydrogenated CoPc as the origin of the observed configurations. In addition, we can rule out atomic defects in NbSe_2_ beneath the CoPc molecules as the origin of the observed configuration, because such defects would break the molecular symmetry, which can be clearly visible in the STM images (Figure S7). Our density functional theory (DFT) simulations further confirm the stability of all four configurations, showing that the equilibrium interlayer separation between CoPc and NbSe_2_ is ∼0.31 nm for the α, β, and γ configurations, while is slightly larger (∼0.34 nm) for the δ (Figure S8). This separation plays an important role in determining the magnetic properties of CoPc/NbSe_2_ (Figure S9).

The most striking result of this work is that the scanning tunneling spectroscopy (STS) recorded on the central Co^2+^ ions of CoPc molecules exhibit pronounced variations across the four stacking configurations (Figure 3). For a gas-phase CoPc molecule, the highest occupied molecular orbital (HOMO) and the lowest unoccupied molecular orbital (LUMO) are located at approximately −1.0 and 0.9 eV, respectively.^34,36^ In contrast, for CoPc molecules on monolayer NbSe_2_, the HOMO in the α, β, γ, and δ configurations consistently appear at about −0.80 eV, as directly captured from the spatially resolved STS spectra shown in Figure 3c-f. These features indicate that all CoPc molecules acquire a slight positive charge due to interaction with the underlying monolayer NbSe_2_.

**Figure 3:**
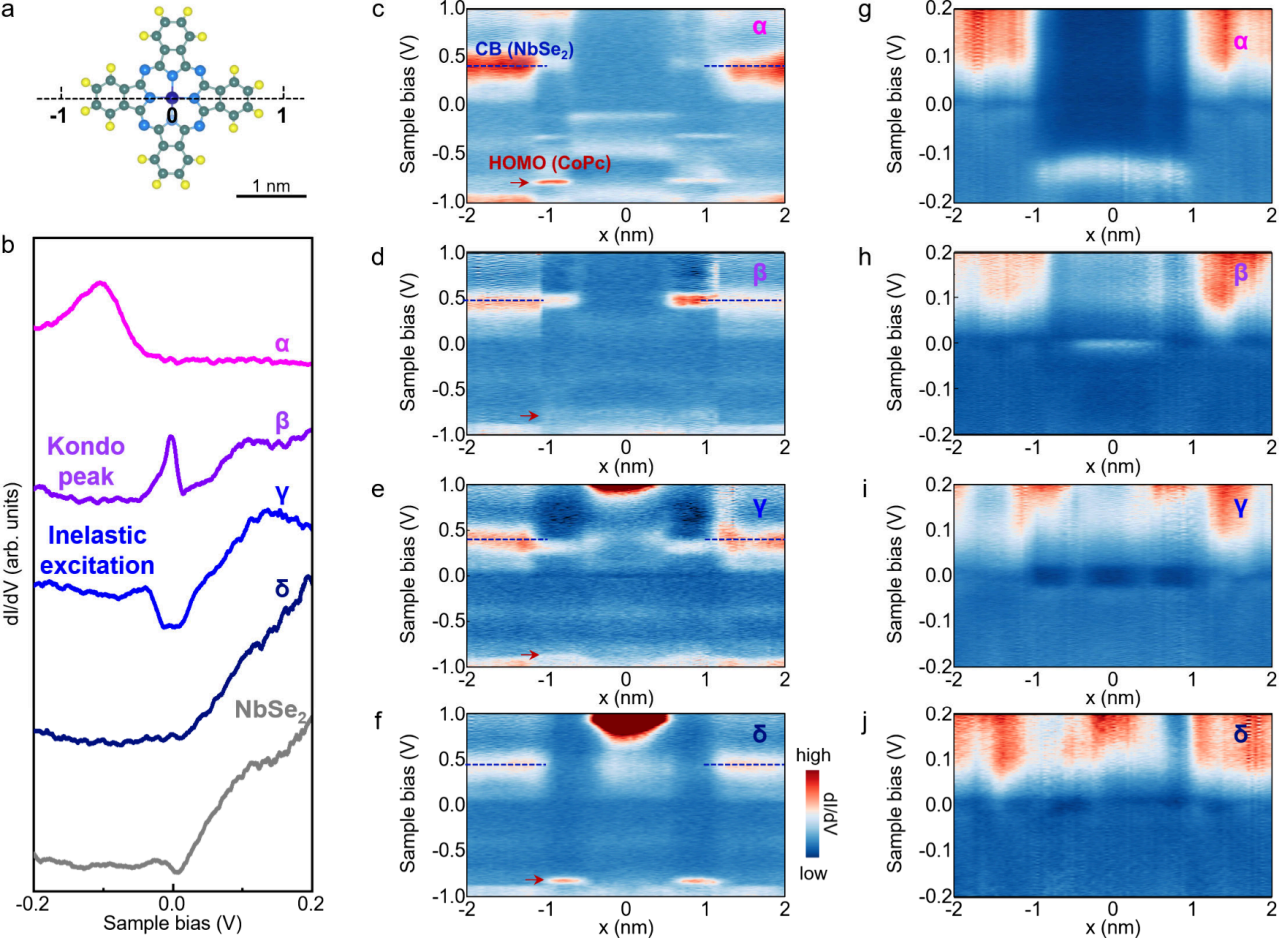
Spectral fingerprint of individual CoPc molecules absorbed on monolayer H-NbSe_2_. (a) Atomic model of an individual CoPc molecule. (b) Representative STS spectra acquired on the bare monolayer H-NbSe_2_ and on the central Co^2+^ ion of CoPc molecules in the α, β, γ, and δ configurations. The spectra are vertically offset for clarity. (c-f) Spatially resolved STS spectra recorded across CoPc molecules in the α, β, γ, and δ configurations along a σ_v_ axis indicated in panel (a). (g-j) Corresponding low-energy STS spectra.

For the positively charged CoPc ad-molecules, the DFT calculations reveal that the ligand spin becomes active and antiparallel to the central Co spin (Figure 1g), owing to the interfacial charge transfer and intramolecular reorganization of the spin density. Meanwhile, the *d*_*Z*_^2^ orbital occupation of the Co remains almost unchanged, resulting in a reduced total magnetic moment in CoPc. This behavior contrasts with that calculated of negatively charged CoPc, where the *d*_*Z*_^2^ orbital becomes more populated, although the molecular magnetic moment is also reduced (Figure 1e). Our DFT calculations further demonstrate that there is about 0.1 electron transferred from CoPc molecules to monolayer NbSe_2_ for all the configurations, yielding the molecular magnetic moments of about 0.6 μ_B_.

Despite the similarity in the charge and spin states of CoPc molecules across these four configurations, their low-energy electronic properties differ significantly (Figure 3b,g-j and Figure S10), which underscores the high sensitivity of the spin coupling between CoPc and monolayer NbSe_2_ on the specific adsorption site. On the one hand, the STS spectra of β-CoPc exhibit a pronounced zero-bias peak (Figure 3b,h), which is absent in the spectra of the other configurations. As previously reported, this peak is attributed to the Kondo resonance,^37–39^ which arises from the screening of the localized spin of CoPc by itinerant electrons in the monolayer NbSe_2_. The presence of the Kondo resonance evidences a strong-coupling regime that sufficiently facilitates the spin coupling between the β-CoPc and the electrons in the monolayer NbSe_2_, as schematically illustrated in Figure 1j. By varying the temperature, we determine a Kondo temperature of ∼ 53 K (Figure S11), much higher than that of bulk NbSe_2_ (0.77 K).^20^ This difference likely arises because superconductivity in bulk NbSe_2_ can suppress spin coupling including Kondo effect, whereas monolayer NbSe_2_ is nonsuperconducting under our measurement conditions.

On the other hand, the spectra of γ-CoPc exhibit obvious differential conductance steps at finite bias, symmetrically distributed around the Fermi level (Figure 3b,i), which serves as a clear indication of the inelastic excitation through electron tunneling. Given that a positively charged γ-CoPc molecule hosts an almost intrinsic *S* = 1/2 spin at the central Co site and an additional ligand spin that couples antiferromagnetically, γ-CoPc is expected to be in the singlet ground state. When the energy of tunneling electrons equals or exceeds the singlet–triplet energy gap, the CoPc molecules transition to the triplet excited state, accompanied by an inelastic electron tunneling process, with the step energy corresponding to the singlet–triplet excitation energy. By examining tens of dI/dV spectra and the corresponding second-derivative curves, we extract an excitation energy of ∼25 meV (Figure S12), which is close to that reported for CoPc on bulk NbSe_2_.^20^ These features suggest that γ-CoPc resides in the weak-coupling regime, where the intramolecular interaction of CoPc dominates the spectra (Figure 1i).

Additionally, the STS spectra recorded on α-CoPc exhibit an extra peak at about −0.1 V (Figure 3b,g), which originates from the π orbitals of the Pc ligands and the *d* orbitals of the central Co^2+^ ion (Figure S13). In contrast, the spectra acquired on δ-CoPc are featureless near the Fermi energy (Figure 3j), confirming that it resides in an almost noncoupling regime, where excessive charge transfer and spin coupling are effectively suppressed (Figure 1h). By precisely choosing the positions and orientations of CoPc molecules relative to the atomic lattice and CDW superlattice of monolayer NbSe_2_, we can achieve transitions among the strong-coupling Kondo regime (β-CoPc), the weak-coupling inelastic excitation regime (γ-CoPc), and the noncoupling regime (δ-CoPc). Therefore, we have successfully tuned the spin interactions at the interfaces between CoPc molecules and monolayer NbSe_2_.

To gain deeper insight into the electronic and magnetic symmetries of CoPc molecules on monolayer NbSe_2_, we carry out bias-dependent topographic and spectroscopic measurements. In topography, CoPc molecules in all configurations consistently exhibit a cross-like shape with 4-fold symmetry, irrespective of the bias voltages (Figure 4a,c and Figure S14). In contrast, spectroscopic maps acquired at 0.4 V reveal a pronounced symmetry breaking from 4-fold to 2-fold in α-CoPc and β-CoPc, while γ-CoPc and δ-CoPc maintain their 4-fold symmetry (Figure 4b,d and Figures S15–S17). These results indicate that the asymmetry of α-CoPc and β-CoPc arises from electronic states rather than atomic structures.^40^

**Figure 4:**
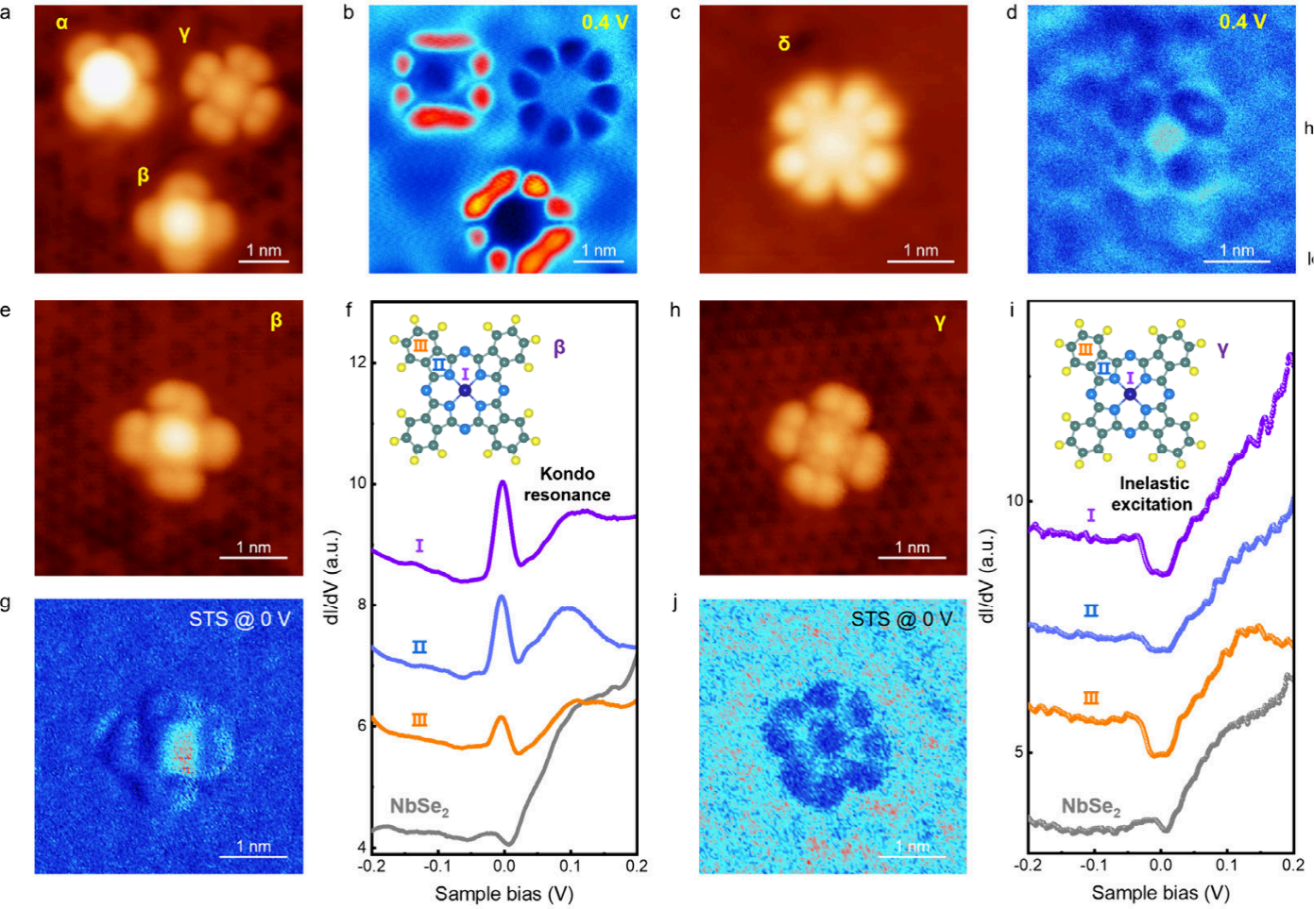
Anisotropy of the charge and spin states of CoPc on monolayer H-NbSe_2_. (a,c,e,h) Representative STM images of CoPc molecules on monolayer H-NbSe_2_ (*V*_*b*_ = −1.0 V, *I*_*t*_ = 10 pA). (b,d) Spectroscopic maps acquired at the same positions as panels (a),(c) under a sample bias of 0.4 V. (f) Site-dependent STS spectra obtained on the β-CoPc and NbSe_2_, with measurement sites indicated in the inset. (g) Zero-bias spectroscopic map acquired at the same position as panel (e). (i) Site-dependent STS spectra obtained on the γ-CoPc and NbSe_2_, with measurement sites indicated in the inset. (j) Zero-bias spectroscopic map acquired at the same position as panel (h).

Such electronic symmetry breaking phenomena are closely linked to magnetic symmetries, as evidenced by our low-energy spectroscopy. To illustrate this, we consider β-CoPc, where electronic symmetry breaking occurs, and compare this case to γ-CoPc, which maintains symmetry. In the site-dependent STS spectra of β-CoPc (Figure 4e,f), the Kondo resonance is observed to span the entire CoPc molecule, with the Kondo peak intensity reaching the maximum at the central Co site. More importantly, the zero-bias spectroscopic map (Figure 4g) reveals that the Kondo-peak intensity decays differently along the two orthogonal directions of β-CoPc, indicating a clear symmetry breaking feature. This is in stark contrast to previous studies of CoPc on metals such as Sb(111), where the Kondo-peak intensity exhibits 4-fold symmetry.^21^ In contrast, the spectroscopic maps acquired at both the Fermi level and the inelastic excitation energy in γ-CoPc display a well-defined 4-fold symmetry, as shown in Figure 4h-j and Figure S18.

Notably, phenomena involving purely intramolecular transitions appear symmetric (γ-CoPc), whereas phenomena arising from the interaction between a magnetic impurity and a metal, such as Kondo screening, clearly exhibit asymmetries (β-CoPc). The latter is plausible to originate from the interplay between the 4-fold symmetry of the adsorbed CoPc molecule and the 3-fold symmetry of the metallic monolayer NbSe_2_, which determines the symmetry of the Kondo active 2D electronic states in the substrate. Our DFT calculations show that the spin density distribution of monolayer NbSe_2_ beneath the β-CoPc exhibits pronounced anisotropy along two perpendicular molecular σ_v_ axes (Figure S19), which may correspond to the anisotropy observed in the Kondo resonance intensity.

Apart from the charge and spin states of CoPc modulated by the underlying monolayer NbSe_2_, the electronic properties of NbSe_2_, conversely, can also be tuned through this coupling. It is worth noting that, in previous studies on Kondo resonances and related phenomena, the influence of magnetic impurities on metallic surfaces has rarely been explored. Figure 5b presents spatially resolved STS spectra acquired on monolayer NbSe_2_ in the vicinity of a γ-CoPc molecule along the trajectory indicated in Figure 5a (more data are given in Figure S20). Remarkably, the dI/dV intensity of the NbSe_2_ conduction band at the energy near 0.5 eV exhibits pronounced real-space oscillations, in stark contrast to the spatially homogeneous charge density distribution of pristine NbSe_2_ (Figure 5c,d).

**Figure 5:**
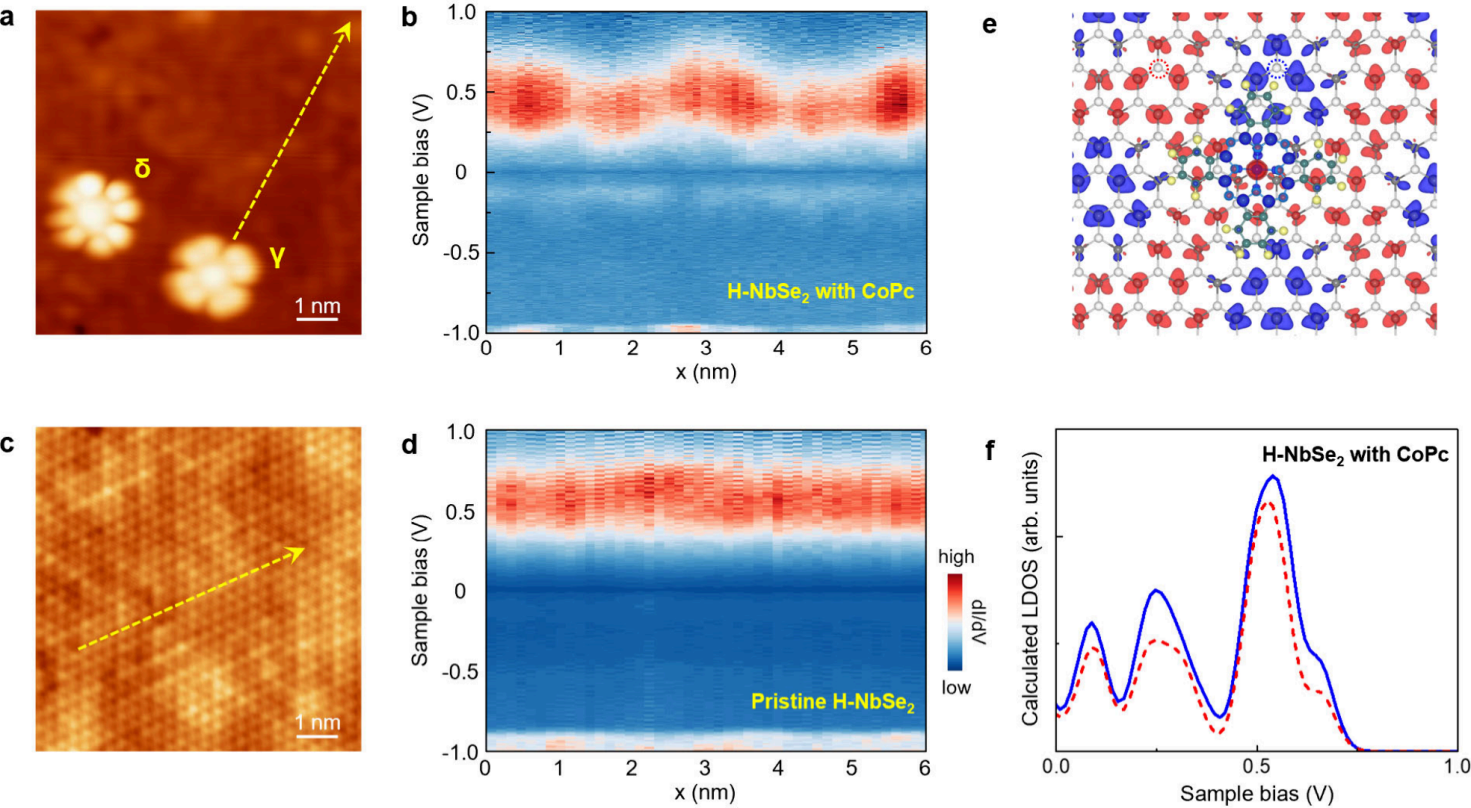
Spin texture of monolayer H-NbSe_2_ induced by the CoPc absorption. (a) Representative STM image of monolayer H-NbSe_2_ with a low dose of CoPc molecules. (b) Spatially resolved STS spectra of NbSe_2_ recorded around a γ-CoPc along the yellow arrow indicated in panel (a). (c) Representative STM image of pristine monolayer H-NbSe_2_. (d) Spatially resolved STS spectra recorded along the yellow arrow indicated in panel (c). (e) DFT calculations of the spin texture in monolayer H-NbSe_2_ induced by γ-CoPc. The red and blue clouds represent spin-up and spin-down electrons, respectively. The charge density isosurface is 0.002e/Å^3^. (f) Theoretical calculations of the LDOS acquired at the locations marked by dotted circles in panel (e).

To elucidate the underlying physics, we perform DFT calculations of monolayer NbSe_2_ coupled with individual CoPc molecules. The spin-density plots in Figure 5e and Figure S21 reveal an anomalous antiferromagnetic order of electron spins in NbSe_2_, distinct from its intrinsic nonmagnetic ground state. Moreover, the calculated local density of states (LDOS) near the γ-CoPc at the spin-up and spin-down regions further demonstrate a significant difference in both energy and intensity (Figure 5f), corresponding to atomic magnetic moments of about −0.13 μ_B_ and 0.09 μ_B_, respectively. These theoretical results well reproduce the measured STS spectra shown in Figure 5b, confirming that CoPc molecules can effectively induce magnetism in monolayer NbSe_2_. However, although the CoPc-NbSe_2_ interaction is stronger in β-CoPc, the magnetism induced in monolayer NbSe_2_ by β-CoPc is experimentally observed to be much weaker (Figure S22), possibly due to the competition between antiferromagnetic order and spin-flip process during Kondo scattering.

## Conclusion

In summary, we demonstrate the ability to manipulate the electronic and spin couplings between CoPc and monolayer NbSe_2_ in a metallic CDW ground state. Four distinct configurations of CoPc on monolayer NbSe_2_ are identified, determined by the relative position of the central Co^2+^ ion with respect to the atomic lattice and the CDW superlattice of NbSe_2_. These configurations not only enable precise modulation of the charge and spin states in CoPc, but also induce magnetism in NbSe_2_. Simultaneously, a pronounced anisotropic Kondo-resonance intensity emerges in CoPc/NbSe_2_. The observed Kondo effect is expected to apply to a wide range of spin-bearing molecules, extending well beyond readily configurable porphyrins and phthalocyanines. Therefore, our findings establish rich tunability of the coupling between molecular spin states and CDW metals. These results open new avenues for engineering complex topological spin textures and for developing molecular spintronic and quantum information devices on demand through atom-precise design of spinterfaces.

## Methods

### Sample Preparation

The BLG was obtained by thermal decomposition of 4H-SiC(0001) at 1220 °C for 40 min. NbSe_2_ layers were epitaxially grown on BLG/SiC(0001) by evaporating Se and Nb from an electron beam evaporator and a Knudsen cell evaporator, respectively. The flux ratio of Se and Nb is more than 10:1 to guarantee a Se-rich environment. The BLG/SiC(0001) substrate was maintained at 500 °C during the growth, followed by a postannealing process at 400 °C for 20 min. MnPc molecules (Sigma-Aldrich) were first purified via a vacuum sublimation, and then were thermally deposited from a Knudsen cell evaporator to NbSe_2_/BLG/SiC­(0001) at 345 °C for 30 min.

### STM/STS Measurements

STM/STS measurements were performed using a commercial low-temperature system (Createc) operated at 4.7 K with a base pressure better than 1 × 10^–10^ mbar. The STM images were taken in a constant-current scanning mode. An electrochemically etched tungsten tip was used as the STM probe, which was calibrated by using a standard graphene lattice and a Si(111)-(7 × 7) lattice. The STS measurements were taken with a standard lock-in technique by turning off the feedback circuit and using a 793-Hz 5 mV A.C. modulation of the sample voltage.

### DFT Calculations

DFT calculations are carried out with Vienna *ab initio* simulation package (VASP)^41^ with the projector augmented wave method (PAW).^42^ The generalized gradient approximation of the Perdew–Burke–Ernzerhof (PBE) form are used to treat exchange-correlation.^43^ The 4*s* ^2^4*p* ^6^4*d* ^4^5*s*,^1^ 4*s* ^2^4*p*,^4^ 3*d* ^8^4*s*,^1^ 2*s* ^2^2*p*,^2^ 2*s* ^2^2*p* ^3^ and 1*s* ^1^ electrons are treated as valence electrons for Nb, Se, Co, C, N and H, respectively. An energy cutoff of 400 eV and Γ-only sampling in the Brillouin zone are used in the geometric relaxation until the energy difference of iteration is smaller than 10^–4^ eV and the force on each atom is smaller than 0.01 eV/Å^2^. A vacuum layer larger than 20 Å is added to avoid interactions between periodical layers. A 9 × 9 supercell of monolayer H-NbSe_2_ is used as the substrate to accommodate the 3 × 3 CDW states of NbSe_2_. DFT-D3 method of Grimme with zero-damping function^44^ is used to treat the vdW interaction between CoPc and NbSe_2_.

## Supplementary Material



## Notes

STM and STS experimental results, molecular structures, DFT calculating results, and more analysis (PDF)
