# Supplementary material for: Tailored Spin Coupling of Single-Molecule Magnets with a Single Charge-Density-Wave Metal Layer
Source: J Am Chem Soc. 2026 Mar 12;148(11):11953–61. doi: 10.1021/jacs.5c21952 (PMC13022852; doi:10.1021/jacs.5c21952)
Supplement: Supplementary file 1 [file ja5c21952_si_001.pdf]

Tailored spin coupling of single-molecule magnets with a single charge-density-wave metal layer

Can Zhang[1,2,†], Fudi Zhou[1,2,†], Heng Jin[3,4,†], Lili Zhou[1,2], Zhaoteng Dong[1,2], Mengya Ren[1,2], Quanzhen Zhang[1], Huixia Yang[1], Xiaolong Xu[1], Yuan Xiao Ma[1], Lan Chen[5,6,7], Thomas A. Jung[8], Bing Huang[4,*], Hong-Jun Gao[5], Yu Zhang[1,2,9,*], Yeliang Wang[1,*]

[1]School of Integrated Circuits and Electronics, MIIT Key Laboratory for Low-Dimensional Quantum Structure and Devices, Beijing Institute of Technology, Beijing 100081, China.

[2]School of Interdisciplinary Science, State Key Laboratory of Environment Characteristics and Effects for Near-space, Beijing Institute of Technology, Beijing 100081, China.

[3]School of Physics, University of Electronic Science and Technology of China, Chengdu 610054, China.

[4]Beijing Computational Science Research Center, Beijing, 100193, China.

[5]Institute of Physics, Chinese Academy of Sciences, Beijing 100190, China.

[6]School of Physical Sciences, University of Chinese Academy of Sciences, Beijing, China

[7]Songshan Lake Materials Laboratory, Dongguan, China

[8]Laboratory for X-ray Nanoscience and Technologies, Paul Scherrer Institut (PSI), 5232 Villigen, Switzerland.

[9]Key Laboratory of Multiscale Spin Physics (Ministry of Education), Beijing Normal University, Beijing 100875, China.

[†]These authors contributed equally to this work.

[*]Correspondence and requests for materials should be addressed to Bing Huang (Bing.Huang@csrc.ac.cn), Yu Zhang (e-mail: yzhang@bit.edu.cn), and Yeliang Wang (e-mail: yeliang.wang@bit.edu.cn).

Table S1: Summarization of the differences between monolayer and bulk $NbSe_2$ and their coupling with CoPc molecules.

	**Monolayer H-$NbSe_2$**	**Bulk 2H-$NbSe_2$**
**Atomic symmetry**	Out-of-plane mirror symmetry<br>No in-plane inversion symmetry	Inversion symmetry
**Fermi surface**	Spin polarization<br>spin-momentum locking	Spin degenerate<br>No spin-momentum locking
**Band structure**	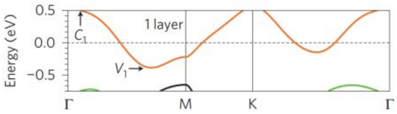	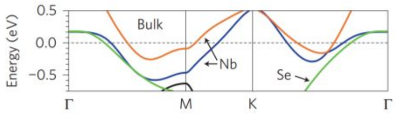
**CDW**	$T_c \sim 145$ K<br>pronounced periodic potential	$T_c \sim 33$ K<br>likely a uniform metal
**SC**	$T_c \sim 3.1$ K<br>Ising superconductor	$T_c \sim 7.2$ K<br>Type II superconductor
	**CoPc adsorption**	
**Structure**	4 configurations	2 configurations
	Dependent on atomic lattice and CDW motif of $NbSe_2$	Dependent on atomic lattice of $NbSe_2$
	Controllable manipulation among the four configurations	no report
Kondo	Kondo and inelastic excitation share a similar CoPc/$NbSe_2$ orientation	Kondo and inelastic excitation have different CoPc/$NbSe_2$ orientations
	Kondo temperature $T_K \sim 53$ K	Kondo temperature $T_K < 1$ K
	anisotropic resonance intensity	no report
Influence on $NbSe_2$	Induce magnetism in $NbSe_2$	no report

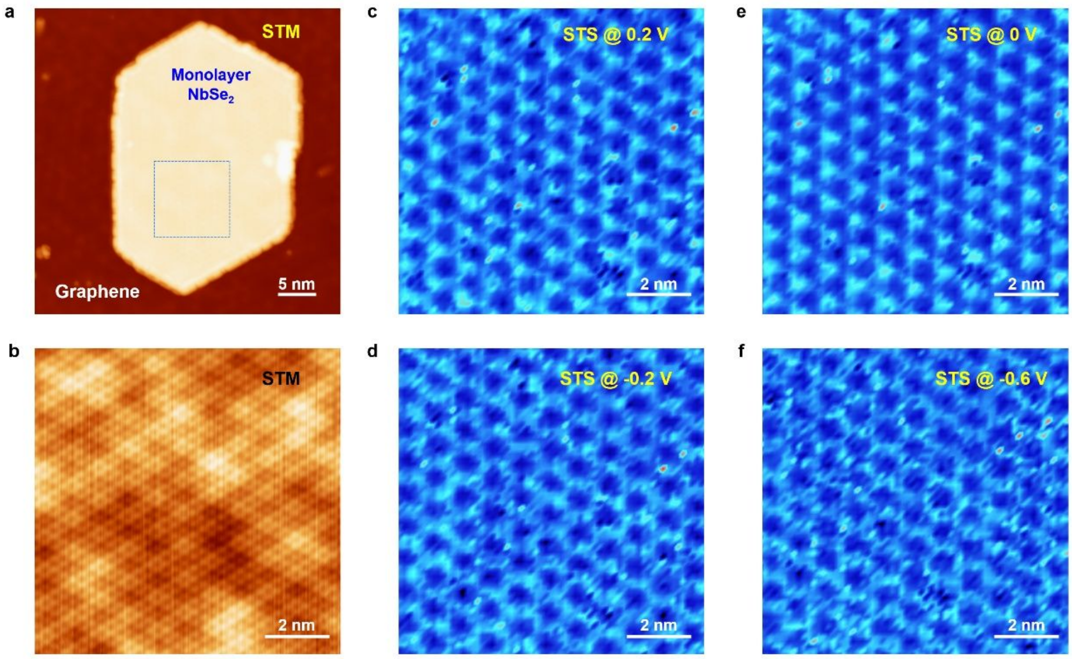

Figure S1. (a) Large-scale STM image of a monolayer H-$NbSe_2$ island on graphene. (b) Atomically resolved STM image of monolayer H-$NbSe_2$ acquired at the position marked in panel a. (c-f) Atomically resolved spectroscopic maps of monolayer H-$NbSe_2$ acquired at the location as panel b under different bias voltages.

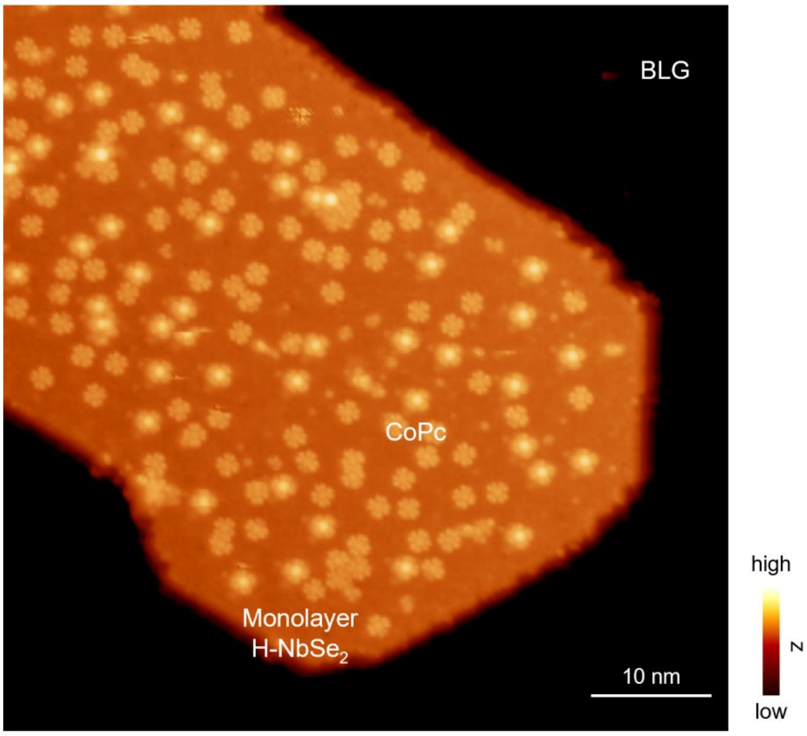

Figure S2. Large-scale topographic STM image of monolayer H-$NbSe_2$ on BLG/SiC(0001) substrates (V_b = -1 V, I_t = 5 pA).

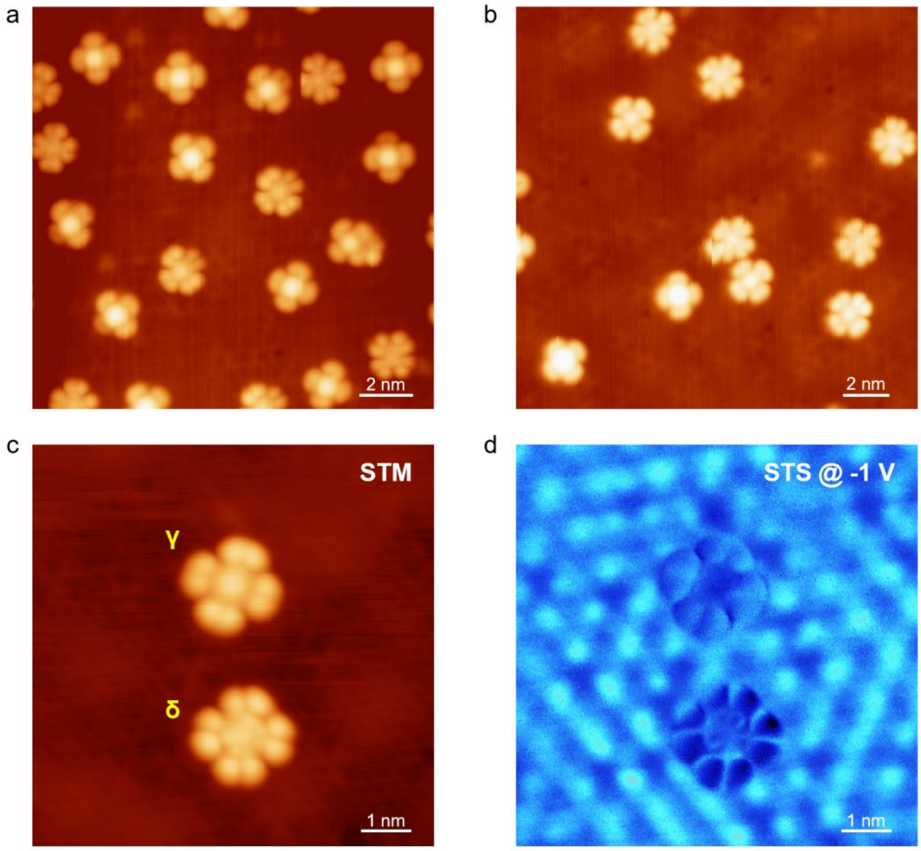

Figure S3. (a,b) Large-scale STM image of CoPc molecules on monolayer H-$NbSe_2$. (c) Representative STM image of CoPc molecules on monolayer H-$NbSe_2$. (d) Spectroscopic map acquired at the same location as panel a under a sample bias of -1 V. The charge density distribution of H-$NbSe_2$ exhibits a periodic modulation, in consistent with the CDW superlattice.

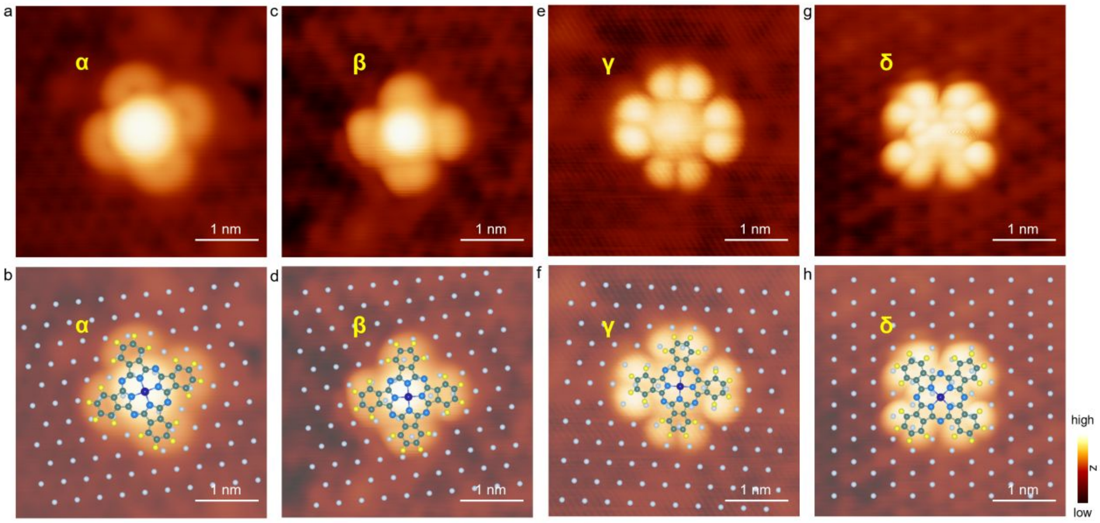

Figure S4. Atomic-resolution STM images of individual CoPc molecules on monolayer H-$NbSe_2$ for the α, β, γ, and δ configurations. (a,b) STM images of α-CoPc. (c,d) STM images of β-CoPc. (e,f) STM images of γ-CoPc. (g,h) STM images of δ-CoPc. The Co^{2+} ions of the CoPc molecules are positioned directly on the topmost Se atoms of monolayer $NbSe_2$ for the α, β, and γ configurations, whereas it is offset from the Se atoms for the δ. The gray spheres indicate the locations of the topmost Se atoms of monolayer $NbSe_2$.

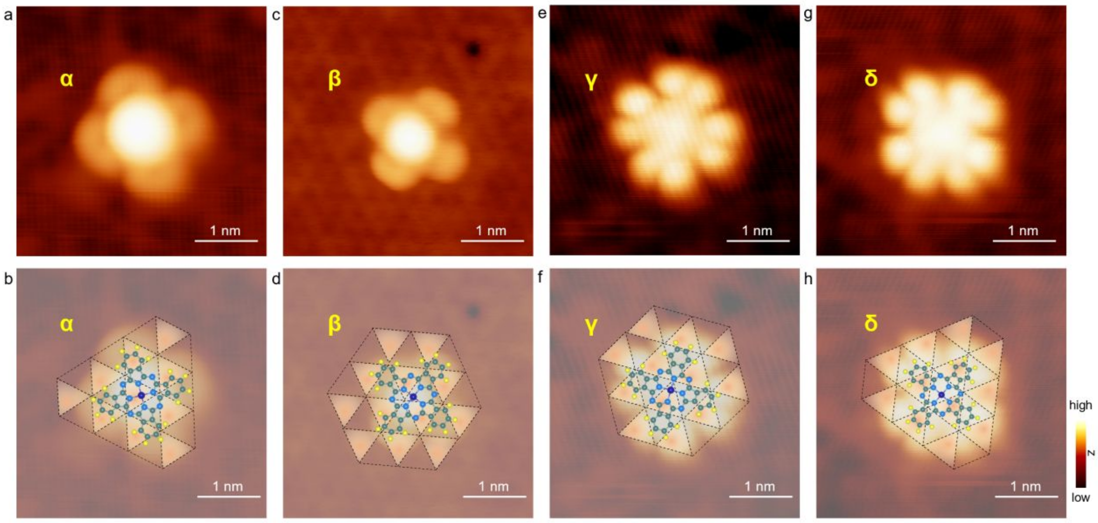

Figure S5. STM images of individual CoPc molecules on monolayer CDW metal H-$NbSe_2$ for the α, β, γ, and δ configurations. (a,b) STM images of α-CoPc. (c,d) STM images of β-CoPc. (e,f) STM images of γ-CoPc. (g,h) STM images of δ-CoPc. The Co^{2+} ion resides on the higher triangular motifs of the CDW superlattice of monolayer $NbSe_2$ for the α and γ configurations, while on the lower motifs for the β and δ.

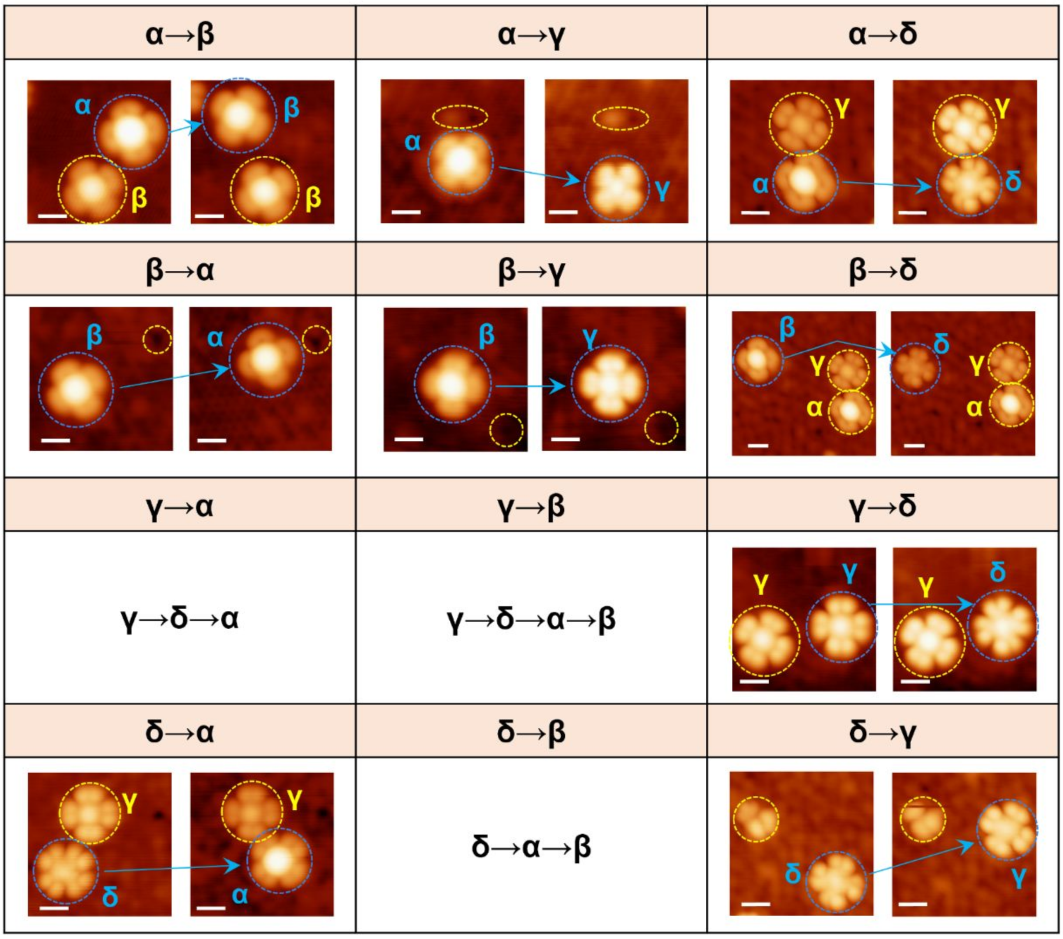

Figure S6. STM images taken during a repositioning sequence. All four configurations can be reversibly switched via the STM tip manipulation. The positions marked by the yellow circles are used for calibration.

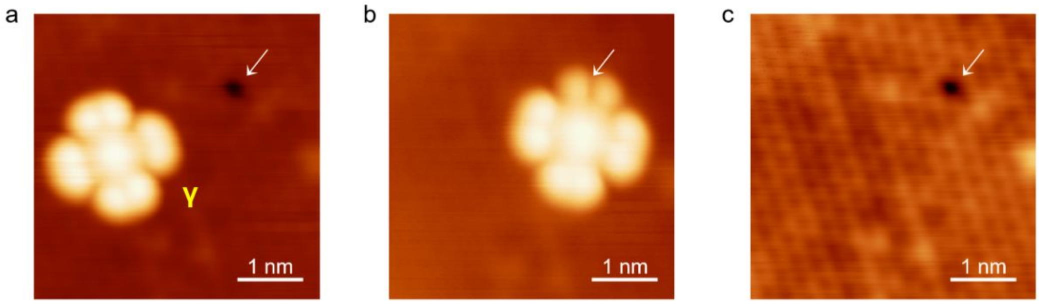

Figure S7. (a) STM image of a γ-CoPc molecule on monolayer H-$NbSe_2$. An atomic defect in H-$NbSe_2$ is indicated by the white arrow. (b) STM manipulation moving the CoPc molecule onto the defect. The lobe positioned above the defect appears clearly distinct from the other three lobes. (c) STM manipulation moving the CoPc molecule out of the field of view. All images are acquired at the same location.

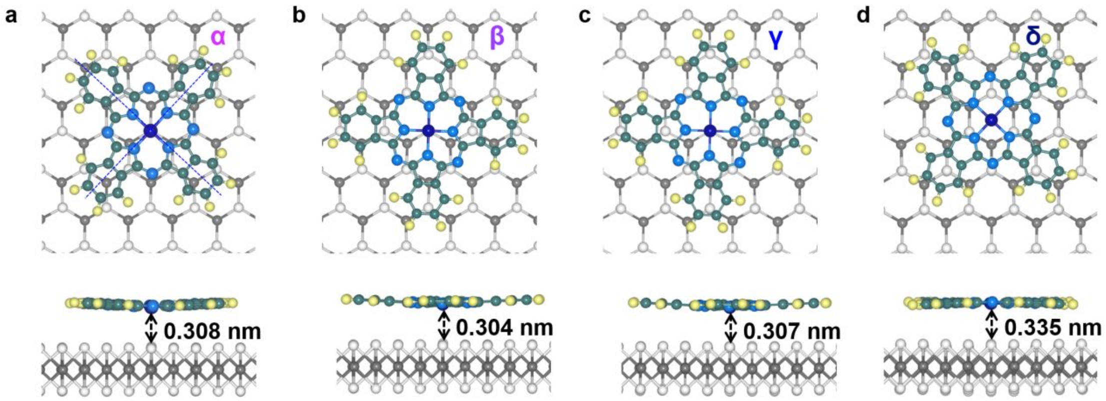

Figure S8. (a-d) DFT calculations of relaxed atomic models for a CoPc molecule on monolayer H-$NbSe_2$ with four distinct configurations. The initial structural parameters are extracted from the measured STM images. Upper panels: top view. Bottom panels: side view. Minor discrepancies are expected across the experiments owing to tip-induced effects, and slightly decreasing of distance (0.018 nm) of a could agree with experiments better.

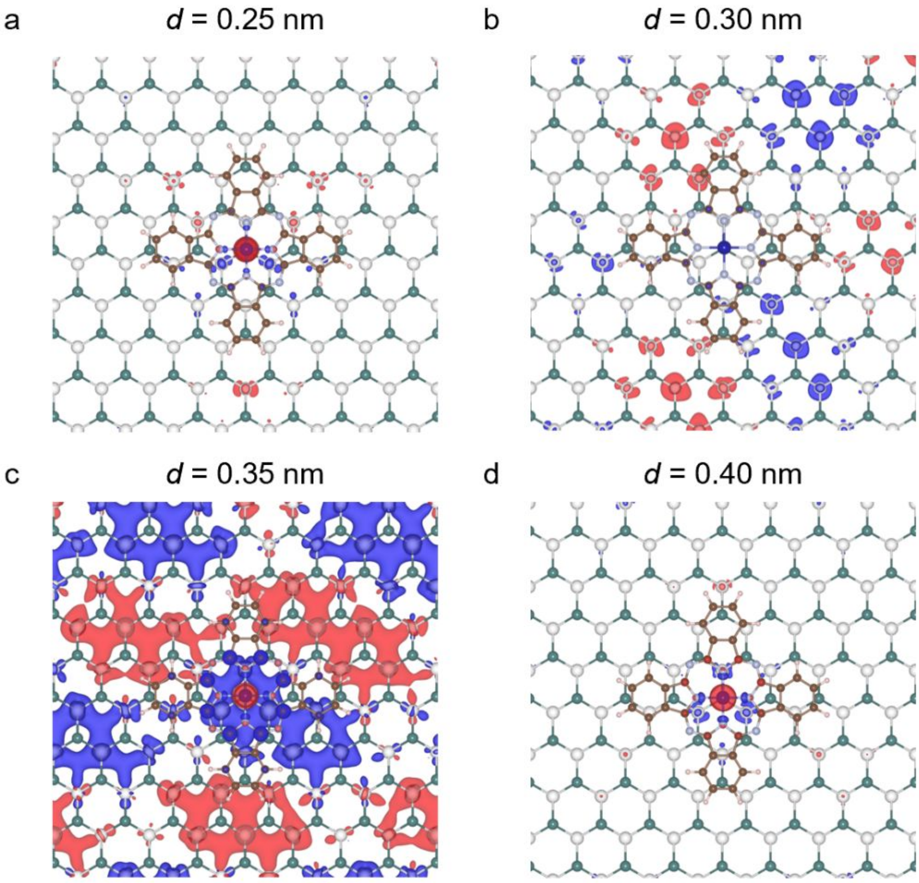

Figure S9. Spin density of the distance-fixed CoPc molecule on monolayer $NbSe_2$ without CDW. The distances between a CoPc molecule and monolayer $NbSe_2$ are fixed at (a) 0.25 nm; (b) 0.30 nm; (c) 0.35 nm; (d) 0.40 nm. The isosurface for all configurations is 0.002 e/$Å^3$.

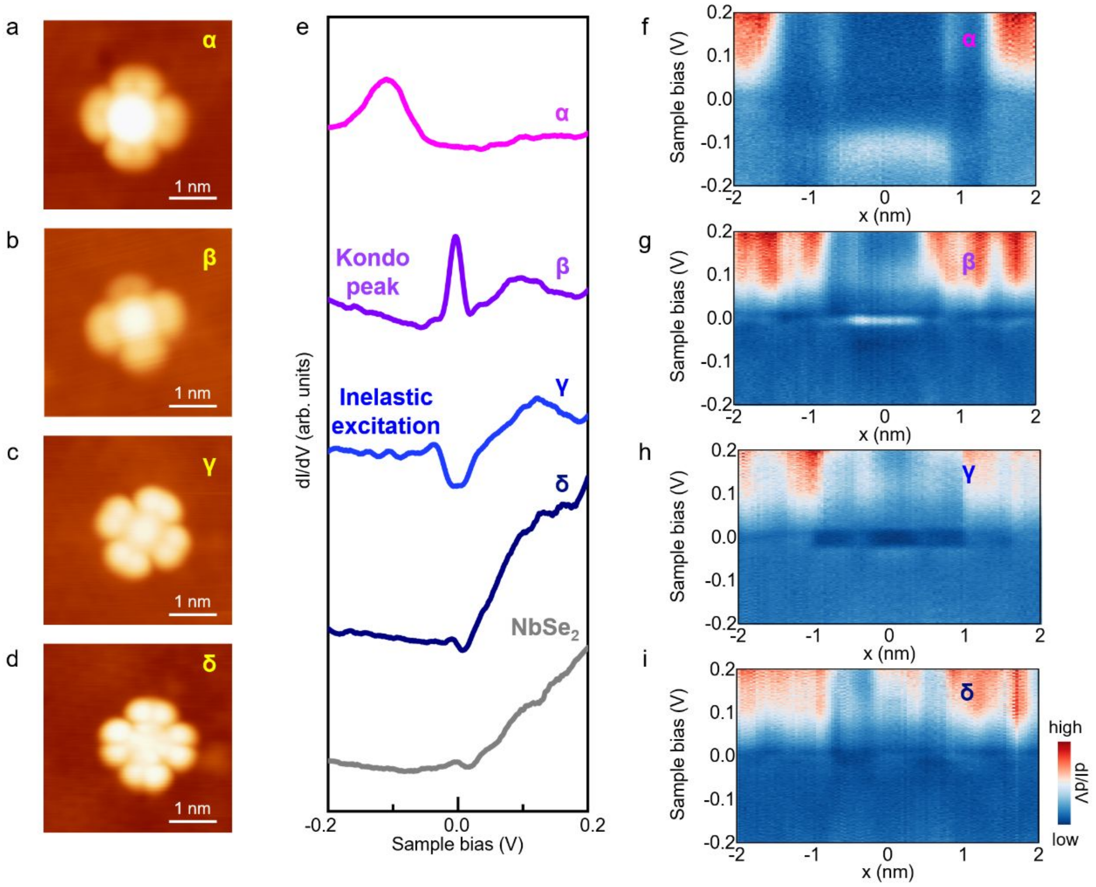

Figure S10. (a-d) STM images corresponding to an additional dataset of spectra acquired from individual CoPc molecules absorbed on monolayer H-$NbSe_2$ in the α, β, γ, and δ configurations, respectively (V_b = -1.0 V, I_t = 10 pA). (e) Representative STS spectra acquired on the bare monolayer H-$NbSe_2$ and on the central Co ion of CoPc molecules in the α, β, γ, and δ configurations. The spectra are vertically offset for clarity. (f-i) Spatially resolved STS spectra recorded across CoPc molecules in the α, β, γ, and δ configurations along the mirror symmetry axis σ_v.

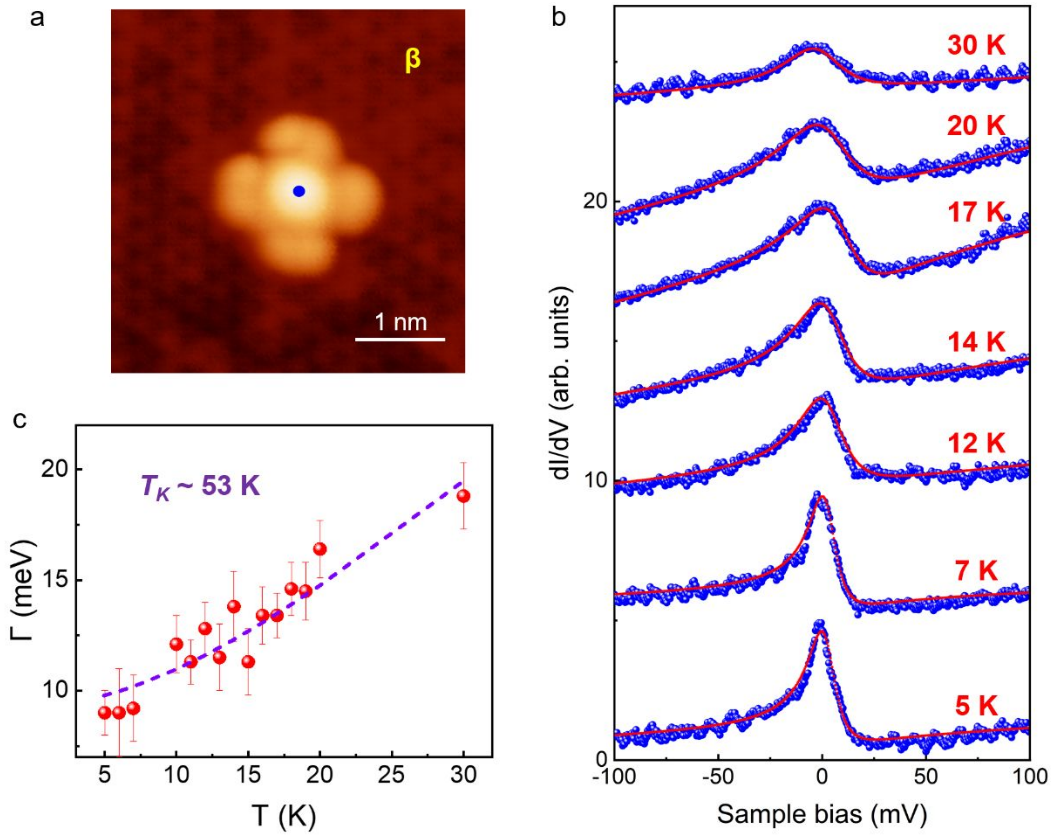

Figure S11. (a) STM image of a β-CoPc molecule. (b) Temperature-dependent dI/dV spectra of the Kondo resonance acquired on the Co^{2+} center of the isolated β-CoPc. The spectra are vertically offset for clarity. (c) Temperature dependence of the Kondo resonance width (red balls) obtained by fitting the dI/dV spectra. The fit (purple line) yields a Kondo temperature of about 53 K.

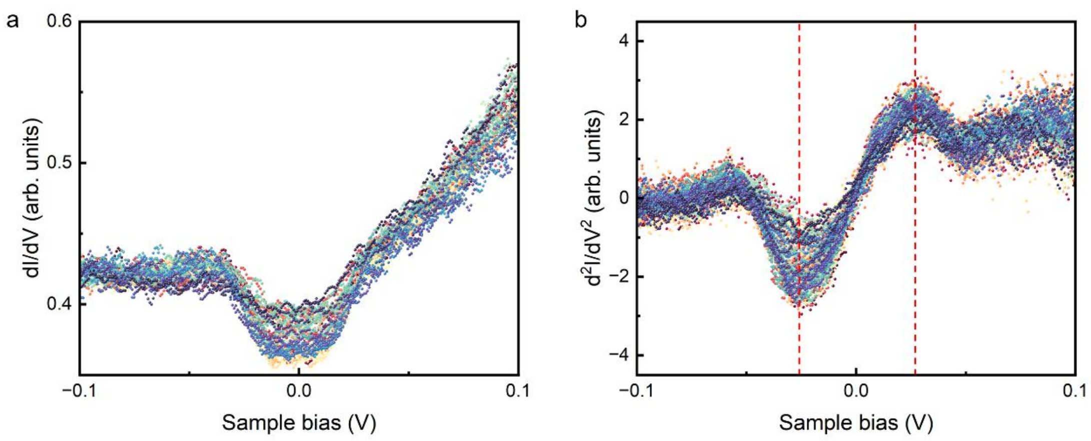

Figure S12. (a) Eighty independent dI/dV spectra acquired on ten distinct γ-CoPc molecules on monolayer H-$NbSe_2$. (b) Corresponding d^2I/dV^2 spectra. The inelastic excitation is observed at a characteristic threshold energy of approximately 25 meV.

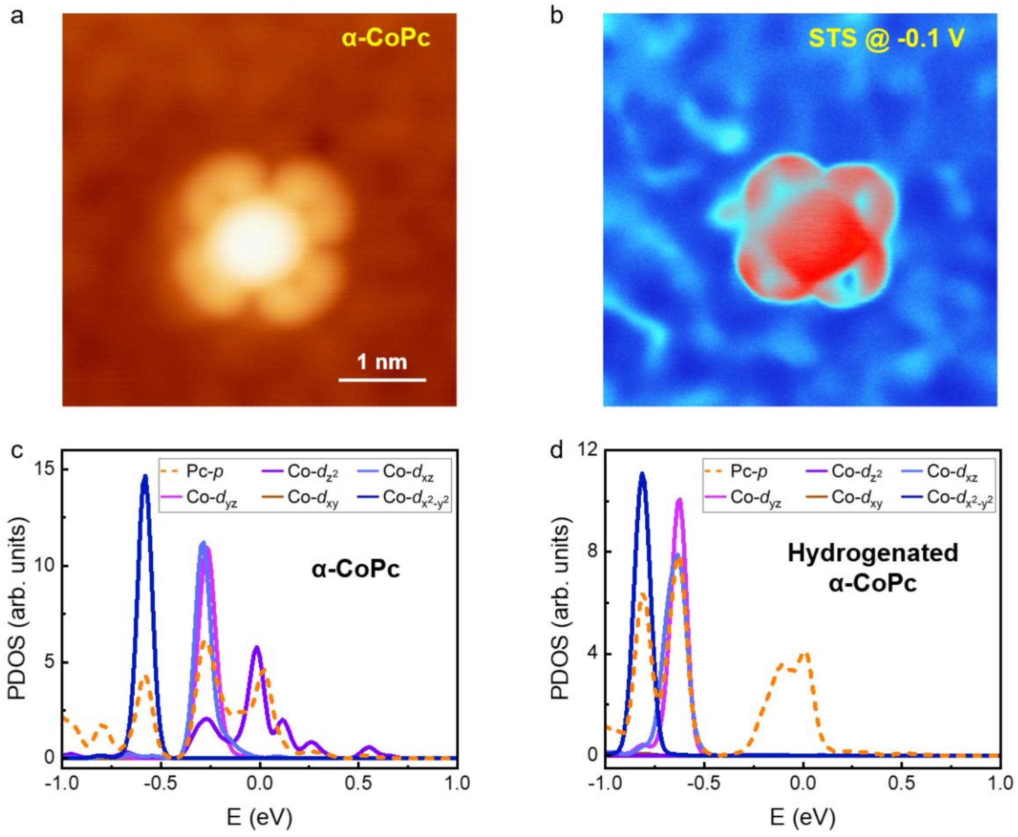

Figure S13. (a) STM image of an α-CoPc molecule on monolayer $NbSe_2$. (b) Spectroscopic map acquired at the same location as panel a under -0.1 V. (c) DFT calculations for α-CoPc on monolayer $NbSe_2$. Both the spectroscopic map and DFT results indicate that the -0.1 V feature originates from the *p* orbitals of the Pc ligands (Pc-*p* orbitals) and the *d* orbitals of the central Co^{2+} ion (Co-*d* orbitals) (d) DFT calculations of a hydrogenated α-CoPc molecule on monolayer $NbSe_2$. The peak at -0.1 V is mainly contributed by the Pc-*p* orbitals, which is inconsistent with the experimental results, allowing us rule out hydrogenation as the origin of the α-CoPc configuration.

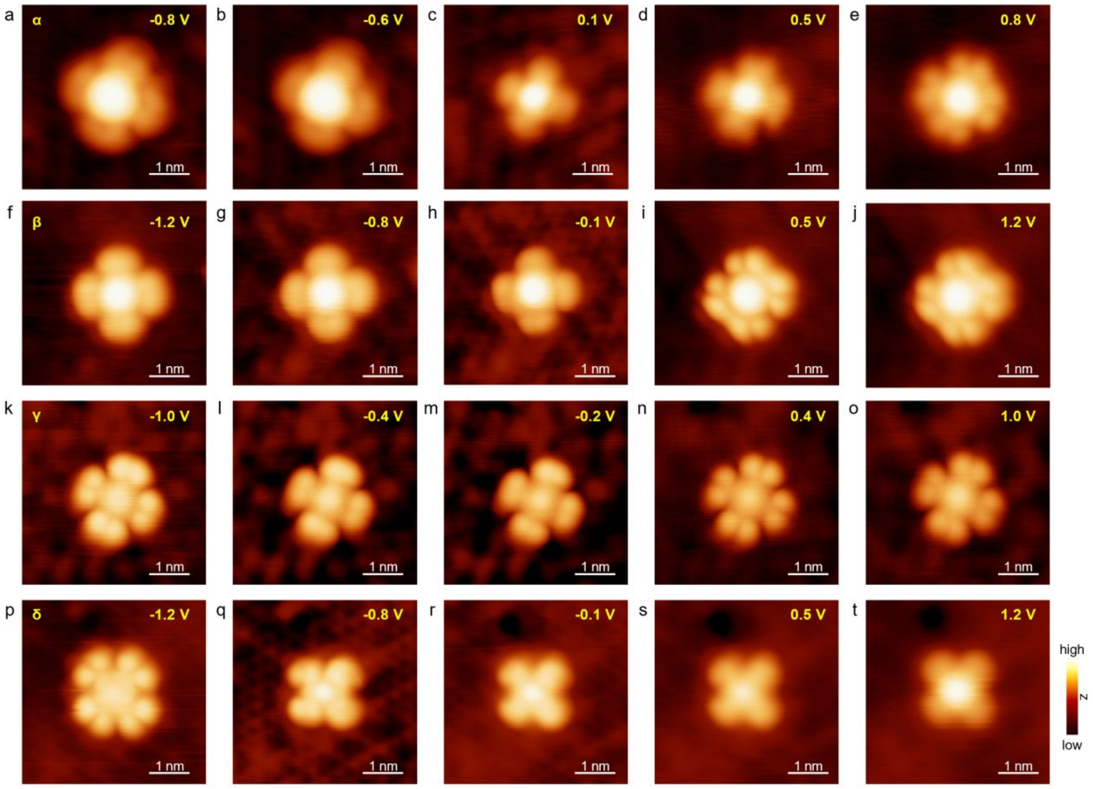

Figure S14. Bias-dependent topographic STM measurements of CoPc molecules on monolayer H-$NbSe_2$ for the α, β, γ, and δ configurations.

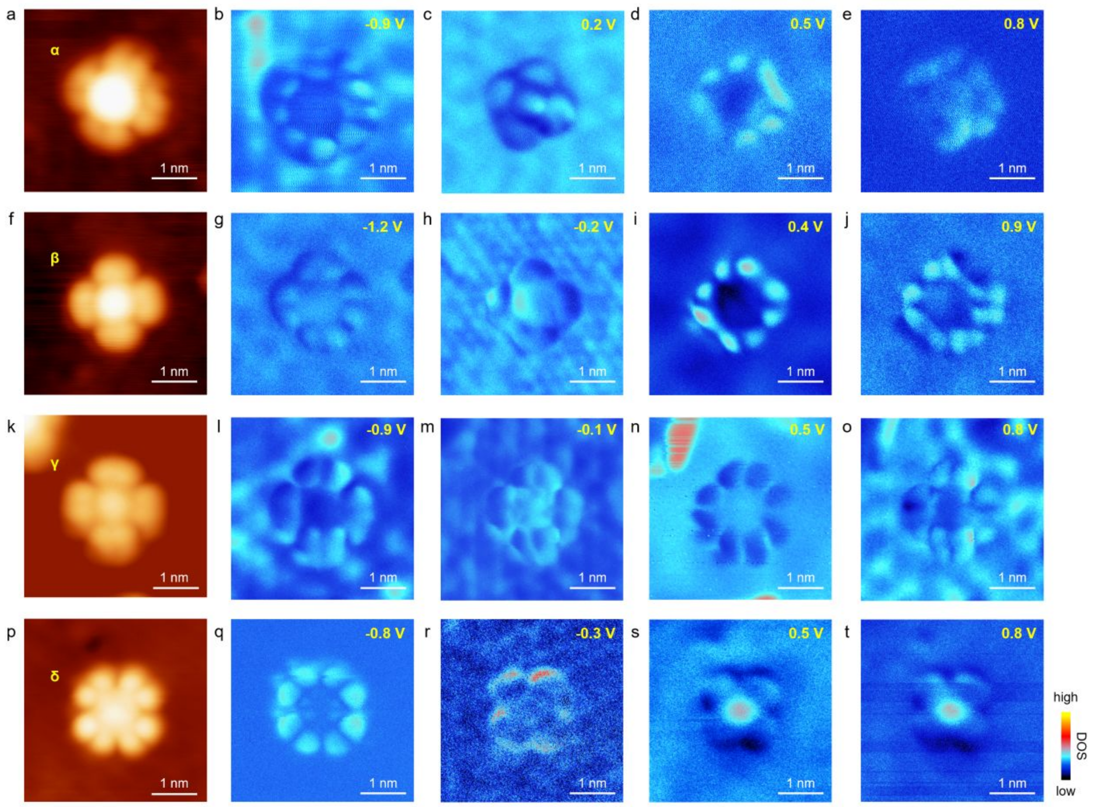

Figure S15. Bias-dependent spectroscopic measurements of CoPc molecules on monolayer H-$NbSe_2$ for the α, β, γ, and δ configurations. (a) STM topographic characteristics of the α-CoPc (V_b = −1.2 V, I_t = 5 pA). (b-e) Spectroscopic maps recorded at the same location as panel a under the sample biases of −0.9 V, 0.2 V, 0.5 V and 0.8 V, respectively. (f) STM topographic characteristics of the β-CoPc (V_b = −1.2 V, I_t = 5 pA). (g-j) Spectroscopic maps recorded at the same location as panel f under the sample biases of −1.2 V, −0.2 V, 0.4 V and 0.9 V, respectively. (k) STM topographic characteristics of the γ-CoPc (V_b = −0.9 V, I_t = 5 pA). (l-o) Spectroscopic maps recorded at the same location as panel k under the sample biases of −0.9 V, −0.1 V, 0.5 V and 0.8 V, respectively. (p) STM topographic characteristics of the δ-CoPc (V_b = −0.8 V, I_t = 5 pA). (q-t) Spectroscopic maps recorded at the same location as panel p under the sample biases of −0.8 V, −0.3 V, 0.5 V and 0.8 V, respectively.

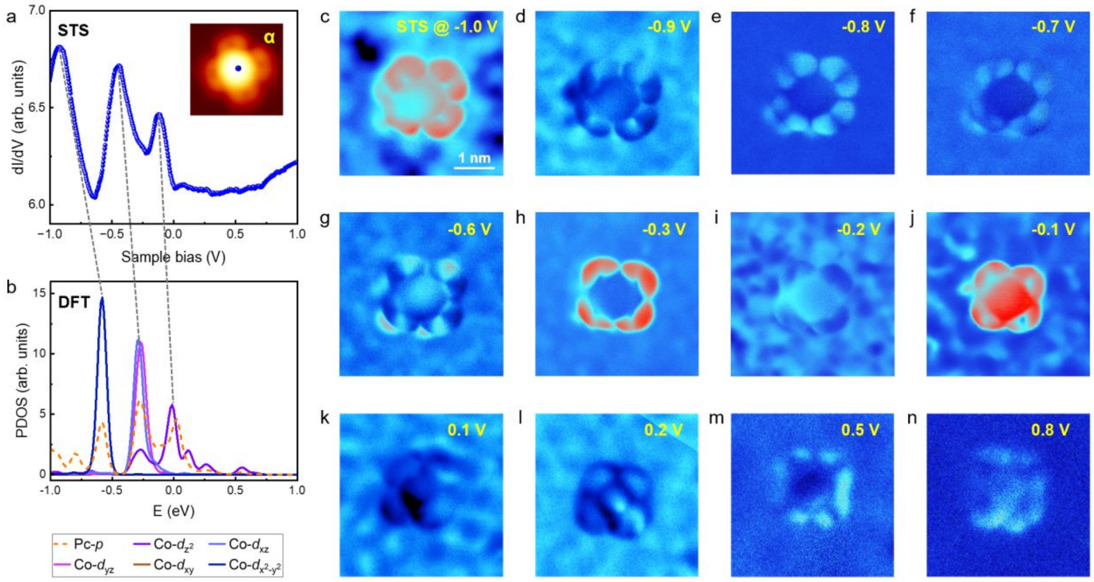

Figure S16. (a) Representative STS spectrum recorded at the Co center of α-CoPc. The mixed *p*-*d* orbitals mainly contribute within the ± 1 V region. (b) Calculated PDOS of α-CoPc. An energy discrepancy is observed between the dI/dV measurements and the DFT results, which may stem from the tendency of the employed exchange-correlation functional to underestimate the band gap, the omission of possible many-body transitions in a molecule, and tip-induced gating effects. (c-n) Spectroscopic maps of a α-CoPc molecule on monolayer H-$NbSe_2$ acquired at different sample biases. The α-CoPc exhibits a pronounced symmetry-breaking behavior near 0.5 V.

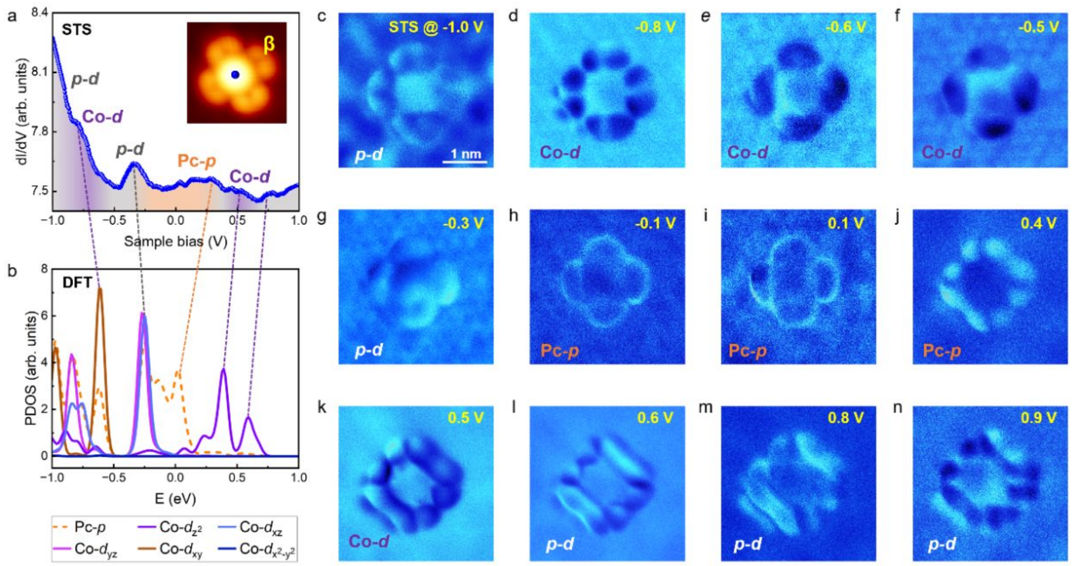

Figure S17. (a) Representative STS spectrum recorded at the center of β-CoPc. The shaded regions in purple, yellow, and gray predominantly correspond to Co-*d*, Pc-*p*, and mixed *p*-*d* orbitals, respectively. (b) Calculated projected density of states (PDOS) of β-CoPc. An energy discrepancy is observed between the dI/dV measurements and the DFT results, which may stem from the tendency of the employed exchange-correlation functional to underestimate the band gap, the omission of possible many-body transitions in a molecule, and tip-induced gating effects. (c-n) Spectroscopic maps of a β-CoPc molecule on monolayer H-$NbSe_2$ acquired at different sample biases. The β-CoPc exhibits a pronounced symmetry-breaking behavior over the bias range of 0.4 to 0.9 V.

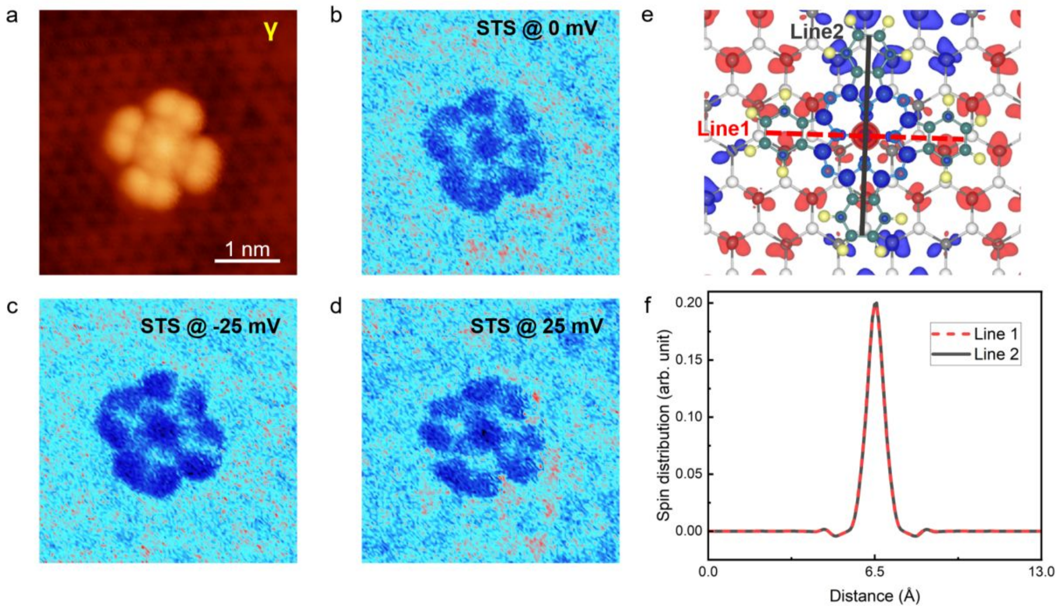

Figure S18. (a) Atomically resolved STM image of a γ-CoPc molecule on monolayer H-$NbSe_2$. (b) Spectroscopic map acquired at the same position as panel a at the Fermi level (0 mV). (c,d) Spectroscopic maps acquired at the same position as panel a under the bias voltages of -25 and +25 meV, corresponding to the excitation energy. All spectroscopic maps in panels b-d exhibit a well-defined fourfold symmetry. (e) Spin density distribution of γ-CoPc absorption on monolayer $NbSe_2$. (f) Spin density distribution of γ-CoPc along the two perpendicular directions of the molecular axes σ_v.

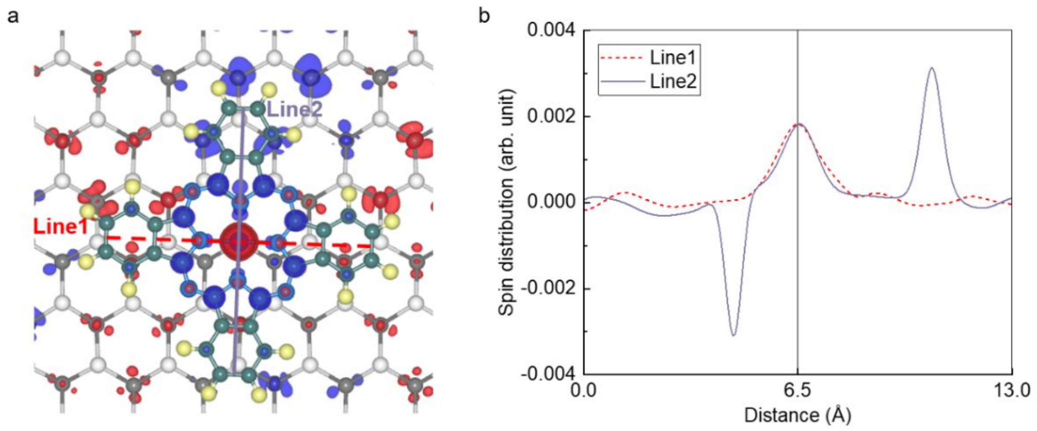

Figure S19. (a) Spin density distribution of β-CoPc absorption on monolayer $NbSe_2$. (b) Spin density distribution of $NbSe_2$ along the two perpendicular directions of the molecular axes σ_v. Such a pronounced anisotropy may correspond to the anisotropy of the Kondo resonance intensity.

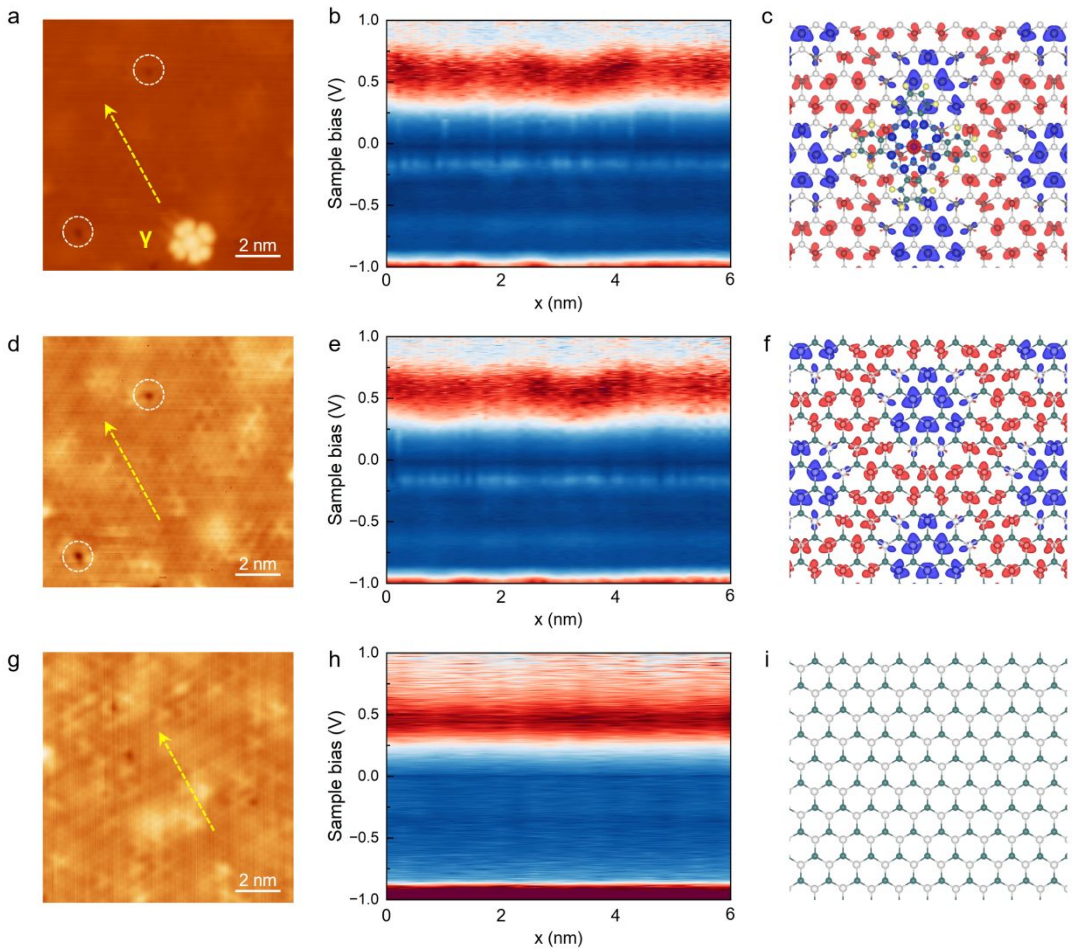

Figure S20. (a) STM image of a γ-CoPc on monolayer $NbSe_2$. (b) Spatially resolved STS spectra recorded around the molecule along the arrow indicated in panel a. The energy of the $NbSe_2$ conduction band exhibits pronounced real-space oscillations. (c) DFT results of the spin texture in monolayer H-$NbSe_2$ induced by γ-CoPc. The red and blue clouds represent the spin-up and spin-down electrons, respectively. The charge density isosurface is 0.002e/Å^3. (d) STM image after removing the molecule via the tip manipulation. The positions marked by the white circles are used for calibration. (e) Spatially resolved STS spectra recorded at the same trajectory. The oscillations remain observable, suggesting that γ-CoPc induces magnetization in monolayer $NbSe_2$. (f) DFT calculations of the spin texture in monolayer H-$NbSe_2$ after removing the γ-CoPc. The magnetism in $NbSe_2$ can be preserved. (g) STM image of monolayer $NbSe_2$ on the same sample, in a region where no molecules had ever been adsorbed. (h) Spatially resolved STS spectra recorded along the arrow indicated in panel g. The charge density is nearly spatially homogeneous, and the slight intrinsic inhomogeneity of $NbSe_2$

surface cannot account for the conduction-band oscillations. (i) DFT calculations of the spin texture of pristine monolayer H-$NbSe_2$, showing no magnetism.

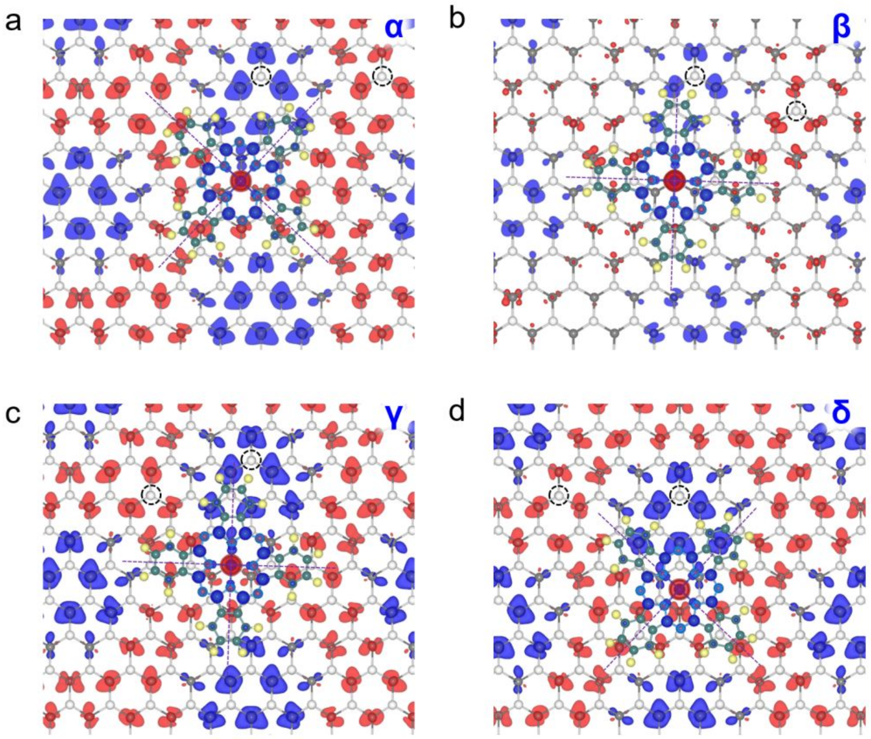

Figure S21. (a-d) Magnetic structures of monolayer H-$NbSe_2$ induced by the absorbed CoPc molecules for the α, β, γ, and δ equilibrium configurations. The charge density isosurface for all these configurations is 0.002e/Å^3. The red and blue clouds represent the spin-up and spin-down electrons, respectively.

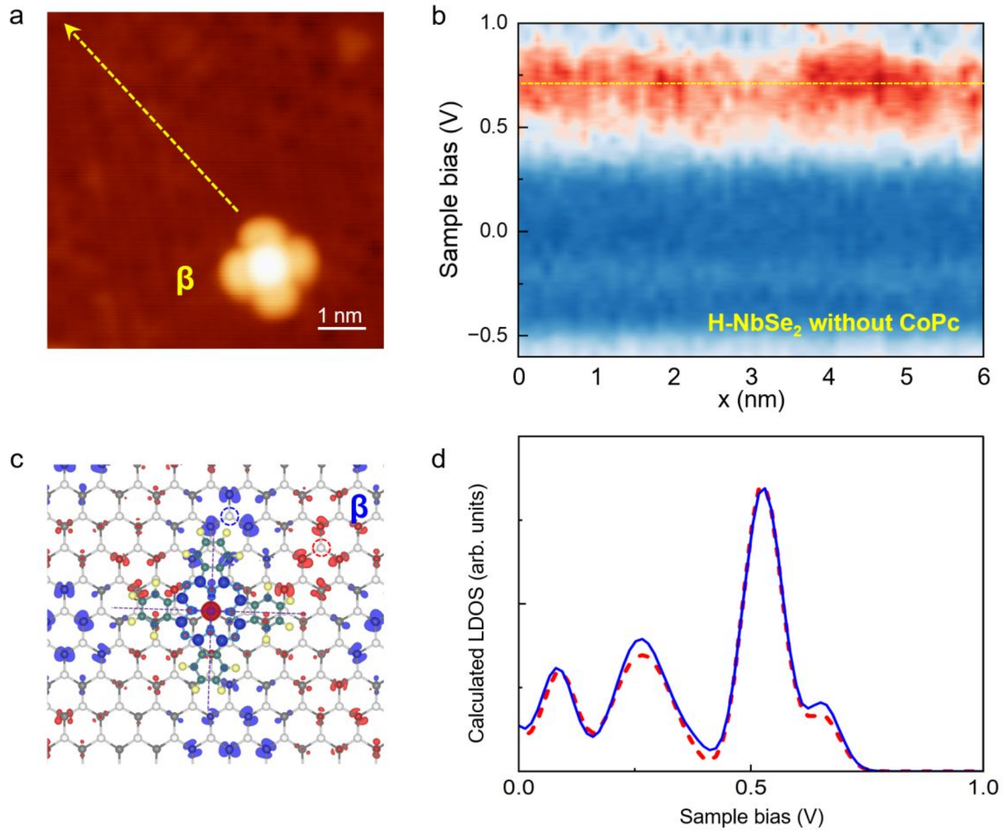

Figure S22. (a) STM image of a β-CoPc on monolayer H-$NbSe_2$. (b) Spatially resolved STS spectra recorded around the β-CoPc molecule along the yellow arrow indicated in panel a. The dI/dV intensity of the $NbSe_2$ conduction band at the energy near 0.5 eV exhibits no obvious oscillation. (c) DFT calculations of the spin texture of monolayer H-$NbSe_2$ induced by β-CoPc. The red and blue clouds represent the spin-up and spin-down electrons, respectively. The charge density isosurface is 0.002e/$Å^3$. (d) Theoretical calculations of the LDOS acquired at the locations marked by dotted circles in panel c.
